# RBBP4: A novel diagnostic and prognostic biomarker for non‐small‐cell lung cancer correlated with autophagic cell death

**DOI:** 10.1002/cam4.70090

**Published:** 2024-08-07

**Authors:** Yajing Zhan, Zhiqian Zhang, Ankang Yin, Xiyang Su, Nan Tang, Yi Chen, Zebin Zhang, Wei Chen, Juan Wang, Wei Wang

**Affiliations:** ^1^ School of Medical Technology and Information Engineering, Zhejiang Chinese Medical University Hangzhou Zhejiang China; ^2^ Department of Clinical Laboratory Center Shaoxing People's Hospital (Shaoxing Hospital) Shaoxing Zhejiang China; ^3^ Department of Laboratory Medicine The Second Affiliated Hospital of Zhejiang Chinese Medical University Hangzhou Zhejiang China; ^4^ Department of Clinical Laboratory People's Hospital of Wangcheng District Changsha Changsha Hunan China; ^5^ Institute of Clinical Medicine Research, Zhejiang Provincial People's Hospital, Hangzhou Medical College Hangzhou Zhejiang China; ^6^ Cancer Institute of Integrated Tradition Chinese and Western Medicine, Zhejiang Academy of Traditional Chinese Medicine Tongde Hospital of Zhejiang Province Hangzhou Zhejiang China; ^7^ Department of Clinical Laboratory, Key Laboratory of Cancer Prevention and Therapy Combining Traditional Chinese and Western Medicine of Zhejiang Province, Zhejiang Academy of Traditional Chinese Medicine Tongde Hospital of Zhejiang Province Hangzhou Zhejiang China

**Keywords:** autophagic cell death, biomarkers, non‐small‐cell lung cancer, prognosis, retinoblastoma‐binding protein 4

## Abstract

**Background:**

Non‐small‐cell lung cancer (NSCLC) often presents at later stages, typically associated with poor prognosis. Autophagy genes play a role in the progression of tumors. This study investigated the clinical relevance, prognostic value, and biological significance of RBBP4 in NSCLC.

**Methods:**

We assessed RBBP4 expression using the GSE30219 and TCGA NSCLC datasets and NSCLC cells, exploring its links with clinical outcomes, tumor immunity, and autophagy genes through bioinformatics analysis after transcriptome sequencing of RBBP4‐knockdown and control PC9 cells. We identified differentially expressed genes (DEGs) and conducted Gene Ontology, Kyoto Encyclopedia of Genes and Genomes pathway enrichment, and protein–protein interaction network analyses. The significance of autophagy‐related DEGs was evaluated for diagnosis and prognosis using the GSE30219 dataset. Experiments both in vivo and in vitro explored the biological mechanisms behind RBBP4‐mediated autophagic cell death in NSCLC.

**Results:**

RBBP4 overexpression in NSCLC correlates with a poorer prognosis. Eighteen types of immune cell were significantly enriched in cultures that had low RBBP4 expression compared high expression. DEGs associated with RBBP4 are enriched in autophagy pathways. Transcriptomic profiling of the PC9 cell line identified autophagy‐related DEGs associated with RBBP4 that exhibited differential expression in NSCLC, suggesting prognostic applications. In vitro experiments demonstrated that RBBP4 knockdown induced autophagy and apoptosis in PC9 cells, promoting cell death, which was inhibited by 3‐MA. In vivo, targeted siRNA against RBBP4 significantly reduced tumor development in PC9 cell‐injected nude mice, elevating autophagy‐related protein levels and inducing apoptosis and necrosis in tumor tissues.

**Conclusion:**

In NSCLC, RBBP4 upregulation correlates with poor prognosis and altered immunity. Its knockdown induces autophagic cell death in NSCLC cells. These results indicate RBBP4 as a potential NSCLC diagnostic marker and its autophagy modulation as a prospective therapeutic target.

## Graphical abstract

RBBP4 is upregulated in NSCLC and its overexpression is linked to poor prognosis and immunity. Its knockdown induces autophagic cell death in NSCLC cells. These findings suggest that RBBP4 may be a potential diagnostic marker for NSCLC; its modulation of autophagy may be a potential therapeutic target

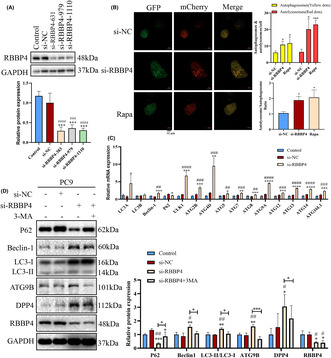

## 1 INTRODUCTION

Lung cancer, the foremost in cancer mortality and second in incidence, accounted for 2.2 million new cases and 1.8 million deaths in 2020.^1^ About 85% of lung cancer instances fall under the non‐small‐cell lung cancer (NSCLC) category.^2^ Despite new antineoplastic drugs, the median survival time for advanced NSCLC patients in the United States has improved by only 1.5 months over more than a decade.^3^ For metastatic NSCLC, the 5‐year survival rate is below 5%.^4^ Most early‐stage NSCLC cases are asymptomatic, leading to late‐stage diagnosis. Thus, there is a pressing need for novel NSCLC diagnostic markers to enhance patient survival rates.^5^

Retinoblastoma binding protein 4 (RBBP4),^6^ located on chromosome 1p35.1, is a member of the highly conserved WD40 repeat protein family, known for its β‐propeller structure that mediates protein–protein interactions.^7^ It plays a crucial role in several complexes, including the Nucleosome Remodeling and Deacetylase (NuRD) complex^8^ and the Polycomb Repressive Complex 2 (PRC2),^9^ where it regulates chromatin remodeling and gene expression through its interaction with the H4α1 helix and H3 tail.^10^ As a core component of the MuvB complex, RBBP4 is also vital in cell cycle regulation.^11^ It is also a regulatory factor in DNA repair,^12^ functioning primarily in the formation of the DNA double helix and the maintenance of a stable chromatin structure.^13^, ^14^ In addition, RBBP4 contributes to telomere stability in tumor cells and supports their unlimited replication.^15^ It governs classical nuclear transport and is involved in cellular senescence.^16^ RBBP4 overexpression has been observed in various cancer types, including hepatocellular carcinoma,^17^, ^18^ acute myeloid leukemia,^19^, ^20^ neuroblastoma,^21^ breast cancer,^22^ thyroid carcinomas,^23^ colon cancer,^24^ and models of embryonal brain tumors.^25^ Furthermore, interfering with RBBP4 expression can significantly inhibit tumor cell growth. In a glioblastoma (GBM) study, knocking down RBBP4 resulted in significant tumor cell sensitization to temozolomide (TMZ) and inhibited multiple DNA repair genes.^12^ Another study suggested the RBBP4/p300 complex is a potential therapeutic target for GBM.^26^ The critical role of RBBP4 highlights its potential as a target for modulating its various biological functions, which could lead to therapeutic applications.

Autophagy, crucial for homeostasis and cell development, is a catabolic process that drives cell degradation and recycling.^27^ Under adverse conditions, autophagy supports cancer cell survival and growth by providing energy and biosynthetic materials,^28^ potentially enhancing their resistance to chemotherapy drugs and thus reducing therapeutic efficacy.^28^, ^29^ While autophagy was originally considered a cell survival pathway, it plays a distinct role in cell death processes.^30^ Studies have suggested that prolonged autophagy, resulting in the overconsumption of cytoplasmic elements, may cause cell death.^31^, ^32^ Although autophagy has been extensively studied in NSCLC,^33^, ^34^ few studies have focused on the multifaceted functions of RBBP4 in this context, particularly the relationship between RBBP4 and autophagic cell death.

In our research, we used datasets from the Cancer Genome Atlas (TCGA) specific to NSCLC and the Gene Expression Omnibus (GEO) to investigate RBBP4's expression and its possible roles in NSCLC. In addition, transcriptomic profiling of PC9 cells was conducted. Based on the results, we constructed a network model of genes differentially expressed (DEGs) in relation to RBBP4 and conducted comprehensive functional and pathway analyses of these genes. Subsequently, we explored the association between the identified DEGs and autophagy genes. In vivo and in vitro validation confirmed the role of RBBP4 in autophagy‐mediated cell death in NSCLC, providing insights into its mechanistic impact on lung cancer cells. Our data can be used to identify novel molecular targets for therapeutic strategies to treat NSCLC.

## 2 MATERIALS AND METHODS

### 2.1 Expression of RBBP4 in NSCLC

We analyzed RBBP4 gene expression in NSCLC using the GEO database (https: //www.ncbi.nlm.nih.gov/; GSE30219, *n* = 286) to collect associated clinical data. We transformed probe‐level data into gene symbols through the use of platform annotation files, with mean values used to represent genes identified with multiple probes. In addition, RBBP4 expression data for lung adenocarcinoma and squamous cell carcinoma were sourced from TCGA using the University of California, Santa Cruz, Xena platform (https://toil.xenahubs.net). This dataset contained 1011 tumor samples and 109 normal samples, including 108 paired samples. After log2 transformation of RBBP4 values, we assessed statistical significance through both Student's t‐test and paired t‐test at a *p*‐value threshold of <0.05.

### 2.2 The association of RBBP4 expression with clinical factors in NSCLC

We investigated how RBBP4 expression correlates with the clinicopathological features of NSCLC patients using the GSE30219 database. RBBP4 expression profiles of NSCLC tissues were compared based on age (<60 or ≥60), sex (male or female), primary tumor (pT) stage (T1, T2, T3, or T4), regional lymph node (pN) stage (N0, N1, N2, or N3), distant metastasis (pM) stage (M0 or M1), and histological subtype. Differences in RBBP4 expression were analyzed using the Wilcoxon test.

### 2.3 Survival analysis

The prognostic value of RBBP4 for NSCLC in the GSE30219 dataset was evaluated using the Kaplan–Meier plotter. We used the R package Survminer (version 0.4.3) to determine the optimal cutoff value, calculated from RBBP4 gene expression, survival time, and survival status. Based on the optimal cutoff value, patients in the GSE30219 dataset with NSCLC were classified into groups with high and low RBBP4 expression. We employed the log‐rank test to determine *p*‐values, considering values below 0.05 as statistically significant for assessing differences.

### 2.4 Gene set enrichment analysis

For a deeper understanding of RBBP4's role, we categorized GSE30219 dataset samples into two groups using the RBBP4 expression cutoff value. We used the ClusterProfiler package (version 3.8.1, http://bioconductor.org/packages/release/bioc/html/clusterProfiler.html) in R software for KEGG pathway enrichment analysis in each subgroup, considering *p* < 0.05 as the threshold for statistical significance.

### 2.5 Roles of RBBP4 in immune regulation

Using the GSE30219 dataset, we conducted single‐sample gene set enrichment analysis (ssGSEA) to assess differences in immune cell enrichment scores across high and low RBBP4 expression groups. This approach assessed the relative level of immune infiltration. Based on previous research, 28 types of immune cell markers were selected.^35^ The Wilcoxon test was employed to identify significant differences in each immune cell type between the groups. We applied Spearman's correlation analysis to investigate the relationship of RBBP4 with variably abundant immune cells.

### 2.6 Analysis of DEGs related to RBBP4 in the GSE30219 dataset and the correlation between RBBP4 and autophagy genes

We utilized the Limma package (Version 3.10.3, http://www.bioconductor.org/packages/2.9/ bioc/html/limma.html) for DEG identification among two expression groups in the GSE30219 dataset (DEGs‐GSE30219). *p* < 0.05 and |logFC| > 0.585 were used as the thresholds for statistical significance. To identify autophagy‐related DEGs (AR‐DEGs) in the GSE30219 dataset (AR‐DEGs‐GSE30219), we analyzed overlapping genes between autophagy‐related genes and DEGs‐GSE30219. Autophagy‐related genes were sourced from both the Human Autophagy Database (HADb) and previous research.^36^, ^37^ The Pearson correlation coefficient and the corresponding *p*‐value between RBBP4 gene expression and each AR‐DEG‐GSE30219 were calculated and visualized.

### 2.7 Enrichment analysis of DEGs upon RBBP4 interference in PC9 cells

PC9 cells, both with and without RBBP4 gene interference, were subjected to transcriptome sequencing for comparative analysis. We used the Limma package (version 3.36.5) in R software for DEG analysis between the RBBP4 gene knockdown group (si‐RBBP4) and the negative control (si‐NC) group (designated DEGs‐PC9). Thresholds of *p* < 0.05 and |logFC| > 0.585 were used to determine statistical significance. We conducted both Gene Ontology (GO) biological process (BP) functional analysis and KEGG pathway enrichment analysis on DEGs‐PC9 using R software's ClusterProfiler package (version 3.18.0). The simplifyEnrichment package of R software was utilized to process the results of GO BP analysis. *p*.adjust <0.05 indicated a statistically significant difference.

We built a protein–protein interaction (PPI) network through the STRING database (Version 10.0, https://string‐db.org/) and employed Cytoscape (version 3.4.0, http://chianti.ucsd.edu/cytoscape‐3.4.0/) for its visualization. A medium‐confidence PPI score above 0.4 as the threshold.

### 2.8 Screening of AR‐DEGs in PC9 cells

To identify AR‐DEGs between the si‐RBBP4 and si‐NC groups (AR‐DEGs‐PC9), we examined the PPI network's nodal topological characteristics, encompassing degree, betweenness, and closeness. This analysis was conducted using the CytoNCA plugin (version 2.1.6, http://apps.cytoscape.org/apps/CytoNCA). After assessing the top 30 genes for each attribute, we identified key DEGs‐PC9 that differentiate si‐RBBP4 from si‐NC. Then, we calculated the Pearson correlation coefficient, r, and the significance *p*‐value between these key DEGs‐PC9 and autophagy‐related genes. We set criteria of |r| > 0.8 and *p* < 0.05 to analyze autophagy‐related genes associated with key DEGs‐PC9. Subsequently, further screening was performed using the clustering analysis package in R (version 3.18.0) to reveal key DEGs‐PC9 enriched in autophagy‐related pathways. These genes were considered potential indirect autophagy‐related differentially expressed genes (iAR‐DEGs) associated with RBBP4. Adjusted *p* < 0.05 was deemed indicative of significance. In addition, by comparing DEGs from both si‐RBBP4 and si‐NC groups against autophagy genes, we identified overlapping genes as direct autophagy‐related differentially expressed genes (dAR‐DEGs) associated with RBBP4.

### 2.9 Prognostic value of AR‐DEGs of RBBP4

Following the extraction of iAR‐DEGs and dAR‐DEGs from PC9 transcriptome sequencing, we assessed their expression levels in the GSE30219 dataset by comparing the tumor group with the normal group and analyzing the differences between the high and low RBBP4 expression groups. Differential expression of each gene was visualized using box plots, with t‐tests employed to obtain *p*‐values for assessing statistical significance. The association of iAR‐DEGs and dAR‐DEGs expression with the prognosis of NSCLC was illustrated using Kaplan–Meier curves. The R package survminer (version 0.4.3) was employed to determine the optimal cutoff value based on the expression levels of iAR‐DEGs and dAR‐DEGs, survival time, and survival status. We employed the log‐rank test for *p*‐value computation, considering values below 0.05 as statistically significant.

### 2.10 Cell lines and culture conditions

We acquired three NSCLC cell lines PC9, H1299, and A549, along with the MRC‐5 human embryonic lung cell line, from the American Type Culture Collection (ATCC, Manassas, VA, USA). The culture conditions for PC9 and H1299 cells involved Dulbecco's Modified Eagle Medium (DMEM, Gibco, Grand Island, NY) supplemented with 10% fetal bovine serum (FBS, Gibco) in an environment maintained at 37°C with 5% CO_2_. We cultured A549 cells in Ham's F‐12 K medium (Gibco), adding 10% FBS (Gibco), and kept them in a controlled environment at 37°C with a 5% CO_2_ atmosphere. Similarly, MRC‐5 cells were grown in minimum essential medium (MEM, Gibco), enriched with 10% FBS (Gibco), and incubated at 37°C in a 5% CO_2_ environment.

### 2.11 Transfection of small interfering RNA

GenePharma Co. (Shanghai, China) synthesized both RBBP4 small interfering RNA (siRNA) and its negative control. For transfection into PC9 cells, we used Lipofectamine 2000 (Invitrogen, Carlsbad, CA). The process involved adding siRNA to serum‐free medium in 6‐well plates, with a seeding density of 2.0 × 10^5^ cells per well. After 6 h, we refreshed the medium with new culture medium supplemented with FBS. We performed all further experiments more than 24 h post‐transfection. The sequences of RBBP4 siRNA were as follows. RBBP4‐homo‐631, sense: 5′‐CCUUCUAAACCAGAUCCUUTT‐3′ and antisense: 5′‐AAGGAUCUGGUUUAGAAGGTT‐3′; RBBP4‐homo‐979, sense: 5′‐GCUGAAGUGAACUGCCUUUTT‐3′ and antisense: 5′‐AAAGGCAGUUCACUUCAGCTT‐3′; and RBBP4‐homo‐1110, sense: 5′‐GGAUGAAAUAUUCCAGGUUTT‐3′ and antisense: 5′‐ AACCUGGAAUAUUUCAUCCTT‐3.

### 2.12 Cell viability assay

We plated PC9 cells in 96‐well plates at a concentration of 5.0 × 10^3^ cells per well and allowed them to incubate overnight. Subsequently, the medium was replaced with culture media containing various concentrations of the autophagy inhibitor 3‐MA (3‐MA, MCE, HY‐19312) (0 mM, 0.15625 mM, 0.3125 mM), followed by a 24‐h incubation period. We assessed cell viability through the Cell Counting Kit‐8 assay (CCK‐8; Dojindo, Kumamoto, Japan). The absorbance at 450 nm was determined with a MAX II microplate reader (Dynex Technologies, Chantilly, VA). Pretreated PC9 cells were collected and enumerated. Then the cells were mixed with 0.4% trypan blue stain (CAS#: 72–57‐1, Sigma) at a 1:1 ratio and incubated for 2–3 min. Through microscopy, the proportions of stained (dead or damaged) cells and unstained (viable) cells were determined. Results are expressed in terms of cell viability percentage.

### 2.13 Apoptosis analysis

After pretreatment, PC9 cells were harvested and rinsed twice using cold phosphate‐buffered saline (PBS) (Biosharp, BL302A). Subsequently, we stained the cells with annexin V‐fluorescein isothiocyanate and propidium iodide (PI) (BD Biosciences, Franklin Lakes, NJ, USA). Analysis was then conducted using flow cytometry (BD Biosciences, Franklin Lakes, NJ, USA), where the total apoptosis was determined by summing the populations in quadrants Q2 and Q3, representing late and early apoptotic cells, respectively.

### 2.14 Western blotting

We quantified protein concentrations with the BCA Protein Assay Kit (Thermo Fisher, Rockford, IL, USA) following the provided protocol. Cell lysates underwent separation via 8%–15% SDS‐PAGE and were subsequently transferred onto polyvinylidene difluoride (PVDF) membranes (Millipore, Billerica, MA, USA). These membranes were then blocked using 5% nonfat milk in TBST for 2 h at room temperature. Overnight incubation at 4°C with primary antibodies was followed by three TBST washes and a 2‐h room temperature incubation with horseradish peroxidase‐conjugated secondary antibodies (TBST 1:2000). Band detection was performed using the ECL chemiluminescence kit (Thermo Scientific, Waltham, MA, USA) and ChemiDoc XRS system (Bio‐Rad), with GAPDH serving as loading controls.

Anti‐beclin‐1 (3495S), anti‐P62 (16177S), anti‐LC3B (3868S), and anti‐GAPDH (2188S) antibodies were purchased from Cell Signaling Technology (CST); anti‐ RBBP4 (20364‐1‐AP) antibodies were purchased from Proteintech; anti‐DPP4 (ab240897) antibodies were purchased from Abcam; and anti‐ATGSE30219B (COA1840) antibodies were purchased from Cohesion Biosciences.

### 2.15 Quantitative real‐time polymerase chain reaction (PCR)

TRIzol reagent (Invitrogen, Life Technologies, Carlsbad, CA, USA) was used to extract total RNA from PC9 cells. We synthesized first‐strand complementary DNA (cDNA) from this RNA using random primers and the RevertAid First Strand cDNA Synthesis Kit (Thermo Scientific, Waltham, MA, USA). Real‐time quantitative PCR was conducted on a CFX96 Touch Real‐Time PCR Detection System (Bio‐Rad, Hercules, CA, USA) employing SYBR Premeix Ex Taq II (Takara, Dalian, China). GAPDH served as the normalization internal control. The specific primer sequences are listed in Supplementary Table S1.

### 2.16 Measurement of autophagic flux

We cultured PC9 cells, modified to express the mCherry‐EGFP‐LC3B reporter gene, in 60 mm confocal‐specific culture dishes at a density of 1.0 × 10^4 cells/well overnight. Following this, the cells were exposed to specified compounds or a dimethyl sulfoxide vehicle for 6 h. Autophagic flux was then examined using a laser scanning confocal microscope (Olympus FV1000, Tokyo, Japan), with images captured randomly. The green fluorescent protein (GFP)‐tagged LC3 plasmid was purchased from Addgene. The mCherry‐GFP‐tagged LC3B plasmid was provided by Noboru Mizushima (Osaka University, Osaka, Japan). For the mCherry‐GFP‐LC3 system, the average numbers of yellow puncta (autophagosomes) and red puncta (autolysosomes) per cell in the merged images were quantified.

### 2.17 Tumor formation assay in nude mouse model

We sourced a 4‐week‐old male Balb/c nude mice from Shanghai Sippe‐Bk Lab Animal Co., Ltd. (China). Each mouse received a subcutaneous injection of 1.0 × 105 PC9 cells into its right axillary area. After the tumors reached a similar size, we randomly allocated the 10 mice into two distinct groups. When tumor diameters reached 5–7 mm, intratumoral multipoint injections were performed every 3 days with either 5 μL Lipo2000 (control group) or 15 μg si‐RBBP4 (si‐RBBP4 treatment group). We measured tumor sizes at 3‐day intervals, determining volume with the formula: volume = A·B^2^/2, where “A” is the longer and “B” the shorter axis length. At the end of the experiment, the mice were killed and the xenograft tumors were excised, weighed, and photographed. We performed all animal experiments in accordance with protocols reviewed and sanctioned by the Animal Experimentation Ethics Committee at Tongde Hospital of Zhejiang Province.

### 2.18 Histopathological examination

Tumor tissues were histologically examined via hematoxylin and eosin staining. Xenograft tumors were fixed, dehydrated with ethanol, paraffin‐embedded, and then cut into 5 μm thick sections. Following deparaffinization, we stained these sections with hematoxylin and eosin, mounted them, and observed under a light microscope at 200× magnification.

### 2.19 Immunohistochemistry

We conducted immunohistochemical staining on tumor tissue sections from mice, each measuring 5 μm in thickness. Tumor sections were deparaffinized and rehydrated, followed by antigen retrieval at 98°C for 15 min. Then the sections were blocked with 5% bovine serum albumin in 0.01 M PBS for 30 min. Subsequently, they were incubated overnight with primary antibodies against RBBP4 (20364‐1‐ap, Proteintech), P62 (D263941, BBI), Beclin‐1 (11306‐1‐AP, Proteintech), LC3B (18725‐1‐AP, Proteintech), DPP4 (29403‐1‐AP, Proteintech), and ATGSE30219B (D124207, BBI). Then they were incubated with a horseradish peroxidase‐conjugated secondary antibody (8114S; CST) at 37°C for 30 min. We then stained these with a 3,3′‐diaminobenzidine (DAB) solution for 5 min and counterstained with hematoxylin. Microscopic observation of the sections was performed at 200× magnification.

### 2.20 Terminal deoxynucleotidyl transferase‐mediated dUTP nick‐end labeling (TUNEL) assay

Apoptosis in xenograft tumors was assessed using the TUNEL assay (Roche, Basel, Switzerland). Tumor sections were treated with 20 μg/mL proteinase K solution for 20 min. Then they were incubated in 3% H_2_O_2_ solution for 5 min. After incubating the sections for an hour at 37°C in the TUNEL reaction mixture (terminal deoxynucleotidyl transferase: biotin‐labeled dUTP solution = 1:9), we then added the converter peroxidase and continued the incubation in a dark, humidified chamber at the same temperature for 30 min. Then the sections were incubated with DAB chromogen solution for 15 min. Hematoxylin was used to stain the cell nuclei. Tumor sections were observed and photographed under a microscope, with positive apoptotic nuclei appearing brown or brown‐black from DAB staining. We quantified the results by calculating the proportion of cells that exhibited positive staining for apoptosis.

### 2.21 Statistical analysis

All experiments were independently replicated at least three times. We employed GraphPad Prism (version 8; GraphPad, San Diego, CA) for data analysis. The results are expressed as the mean ± standard deviation. Statistical significance was determined by Student's *t*‐test or analysis of variance, with a significance threshold set at *p* < 0.05.

## 3 RESULTS

### 3.1 RBBP4 gene expression in NSCLC

**FIGURE 1:**
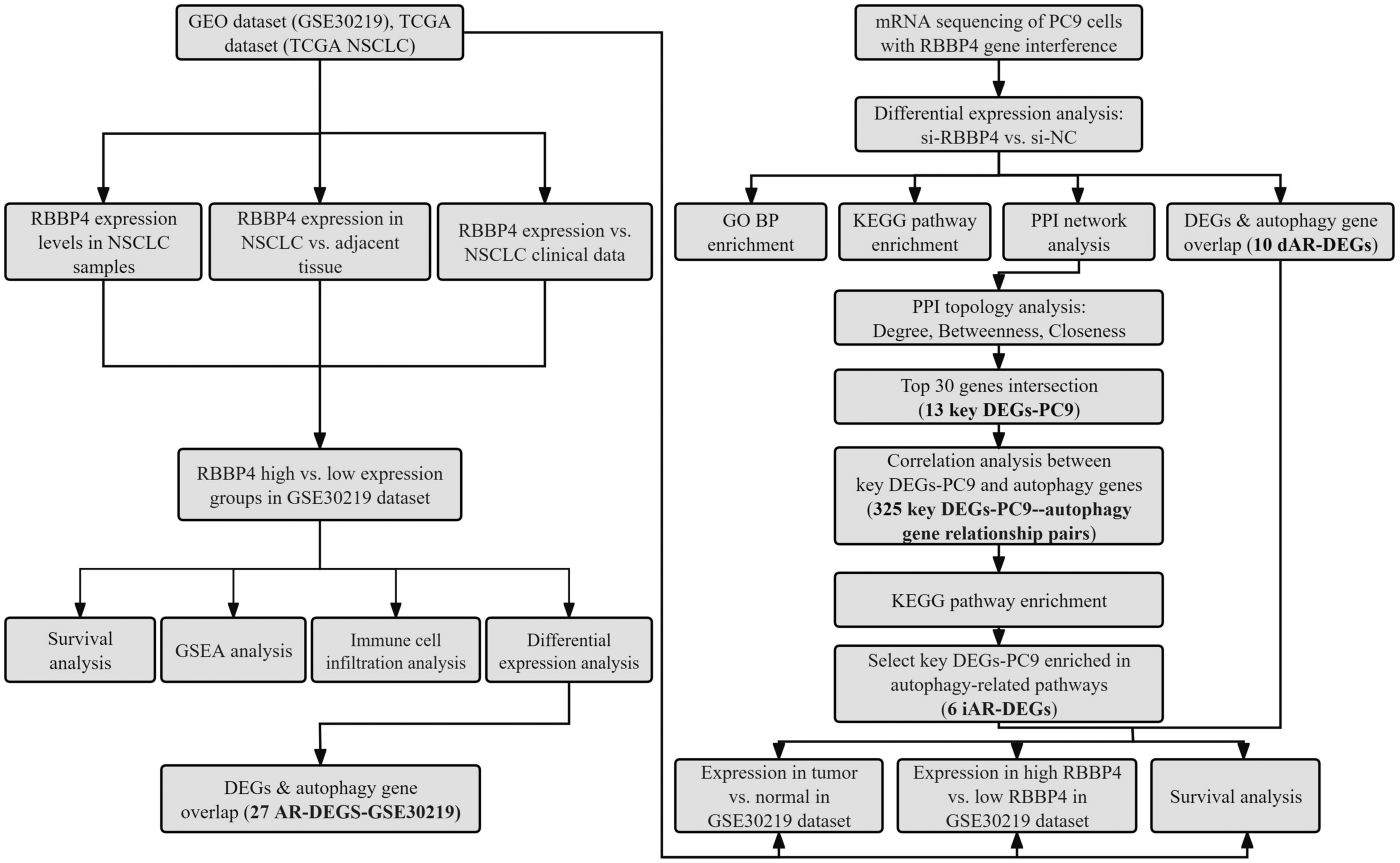
Workflow of comprehensive transcriptome analysis for RBBP4. We assessed the expression levels of RBBP4 using the GSE30219 and TCGA NSCLC datasets, exploring their correlation with clinical data, immune infiltration, and patient prognosis. In addition, we conducted gene set enrichment analysis and identified DEGs associated with autophagy. Subsequently, we performed transcriptome sequencing on RBBP4‐knockdown PC9 cells and control PC9 cells, analyzed differential gene expression and performed GO BP, KEGG, and PPI enrichment analyses, and screened for DEGs related to autophagy. Finally, we investigated the expression levels and prognostic implications of these genes in NSCLC.

**FIGURE 2:**
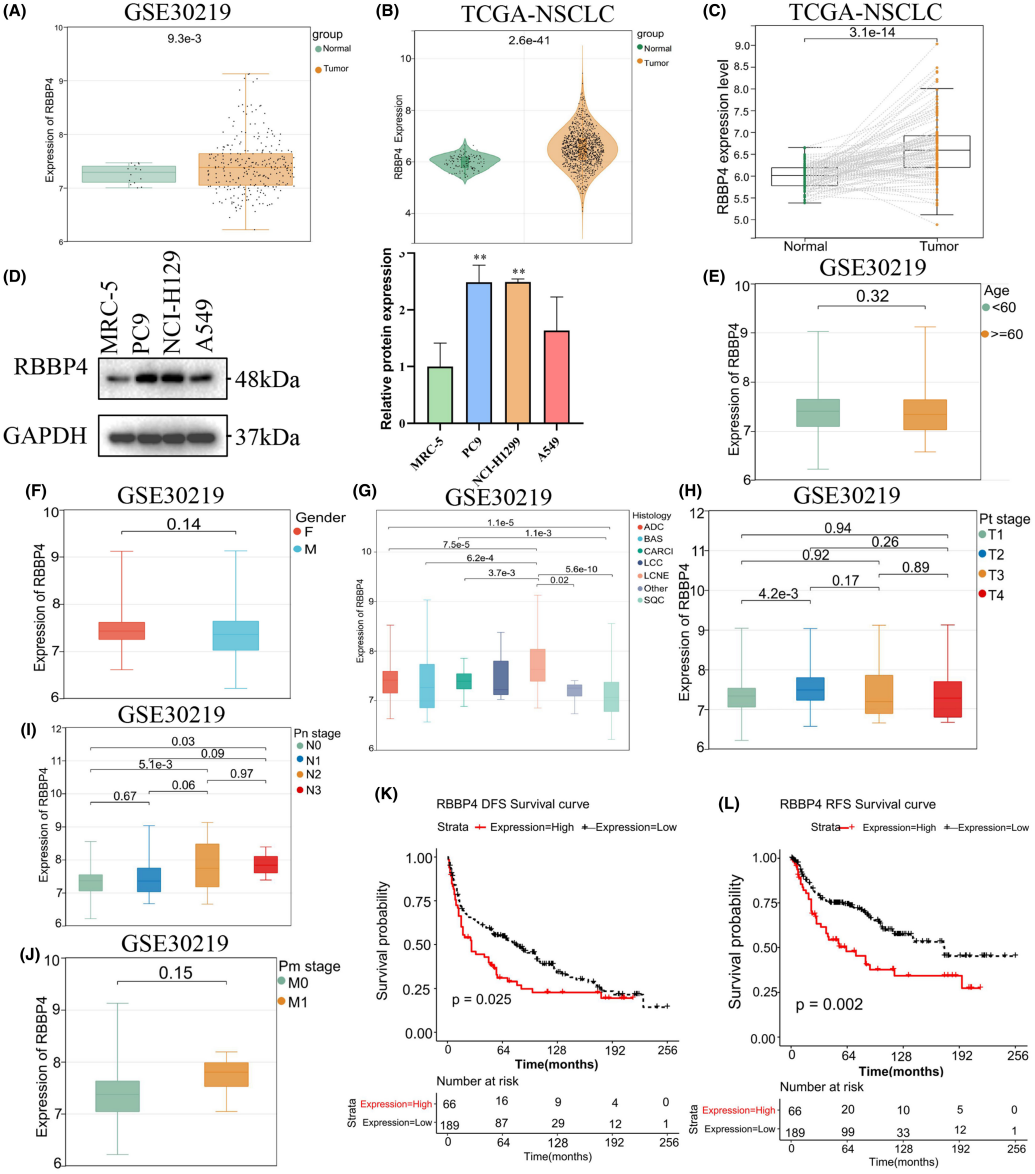
RBBP4 expression, clinical correlation, and prognostic significance in NSCLC. (A) RBBP4 gene expression in the GSE30219 dataset (tumor: *N* = 272; normal: *N* = 14). (B) RBBP4 expression in the TCGA‐NSCLC dataset (tumor: *N* = 1011; normal: *N* = 109). (C) RBBP4 expression in paired cancer and adjacent tissues from the TCGA‐NSCLC dataset (*n* = 108). (D) Comparative assessment of RBBP4 expression levels in MRC‐5, PC9, H1299, and A549 cells, **p < 0.01 versus the MRC‐5 cells. (E–H) Association between RBBP4 expression and clinicopathological features in the GSE30219 dataset: (E) age, (F) gender, (G) histology, (H) pT stage, (I) pN stage, and (J) pM stage. (K) Survival curves of recurrence‐free survival with high (red) and low (black) RBBP4 expression in the GSE30219 dataset using the Kaplan–Meier plotter (high expression: *N* = 66, low expression: *N* = 189). The optimal cutoff between high and low RBBP4 gene expression is 7.591442. (L) Survival curves of disease‐free survival with high and low RBBP4 expression in the GSE30219 dataset using the Kaplan–Meier plotter.

Figure 1 illustrates the study's workflow. We utilized the National Center for Biotechnology Information Gene Expression Omnibus database and TCGA datasets to analyze the differential expression of RBBP4 in NSCLC. RBBP4 expression was significantly elevated in NSCLC samples compared to normal ones in both GSE30219 and TCGA datasets, with *p*‐values of 0.0093 and <0.0001, respectively (Figure 2A,B). Furthermore, we analyzed 108 pairs of cancer and adjacent tissues in the TCGA dataset, and the paired t‐test revealed that RBBP4 expression was significantly upregulated in tumor tissues compared to adjacent noncancerous tissues (*p* < 0.0001) (Figure 2C). These results demonstrate that RBBP4 expression is higher in NSCLC than in adjacent noncancerous tissues. To confirm RBBP4 levels in tumor cells, we investigated RBBP4 expression in three lung cancer cell lines, namely, PC9, H1299, and A549. Western blotting (WB) analysis revealed that, compared to MRC‐5 cells, RBBP4 expression was upregulated in PC9, H1299, and A549 cells. The most pronounced upregulation was observed in PC9 cells (Figure 2D).

We assessed RBBP4's prognostic relevance in NSCLC patients by examining its association with clinicopathological characteristics in the GSE30219 dataset. Statistical analysis revealed that RBBP4 expression levels were significantly elevated in lung adenocarcinoma (ADC) compared to lung squamous cell carcinoma (SQC) (*p* < 0.0001) (Figure 2G). Patients in T2 stage exhibited a significant increase in RBBP4 expression compared to T1 stage (Figure 2H). With the advancement in N stage, a rising trend in RBBP4 expression was observed, particularly in N2 and N3 stage patients, who showed a notable increase compared to N1 stage (Figure 2I). Although RBBP4 expression also increased with higher M stages, this rise did not reach statistical significance (Figure 2J). Age and gender did not lead to significant differences in RBBP4 expression (Figure 2E,F).

### 3.2 High expression of RBBP4 is associated with poor prognosis in NSCLC patients

We evaluated RBBP4's prognostic significance in NSCLC through analysis with the Kaplan–Meier plotter. Based on the GSE30219 dataset, samples were categorized into two groups using the optimal critical value of RBBP4 expression, which was 7.591442. This categorization led to 66 samples being placed in the high‐expression group and 189 in the low‐expression group. The data indicated that patients with high RBBP4 expression had worse recurrence‐free survival (RFS) and disease‐free survival (DFS) than patients in the low‐expression group (Figure 2K,L). These findings suggest that elevated RBBP4 expression may be indicative of a poorer prognosis for NSCLC patients.

### 3.3 Gene set enrichment analysis

**FIGURE 3:**
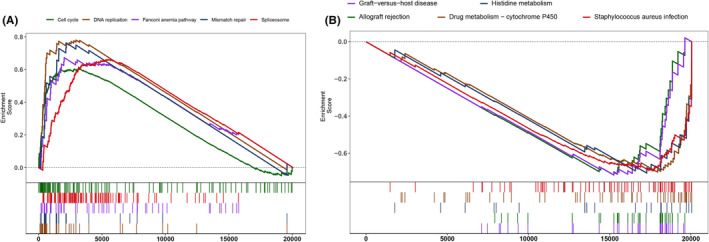
GSEA results for RBBP4 in the GSE30219 dataset. (A) Top five KEGG pathways significantly enriched in the high RBBP4 expression group; (B) top five KEGG pathways significantly enriched in the low RBBP4 expression group.

Using gene set enrichment analysis (GSEA), we explored signaling pathways associated with RBBP4 in NSCLC in the high‐ and low‐expression phenotypes. Twenty KEGG pathways were predominantly represented in the high‐expression group, primarily linked to gene instability, potentially explaining the observation of poor prognosis. By contrast, 95 KEGG pathways were notably prevalent in the group with lower RBBP4 expression. The top five pathways for the high‐expression group that were significant at *p* < 0.05 included biological processes such as cell cycle regulation, DNA replication, the Fanconi anemia pathway, mismatch repair, and spliceosome‐mediated mRNA splicing (Figure 3A). By contrast, the top five pathways for the low‐expression group were associated with graft‐versus‐host disease, histidine metabolism, allograft rejection, drug metabolism via cytochrome P450, and *Staphylococcus aureus* infection (Figure 3B). These findings elucidate the distinct molecular mechanisms between high and low RBBP4 expression groups.

### 3.4 Analysis of the immunomodulatory effects of RBBP4 in NSCLC

**FIGURE 4:**
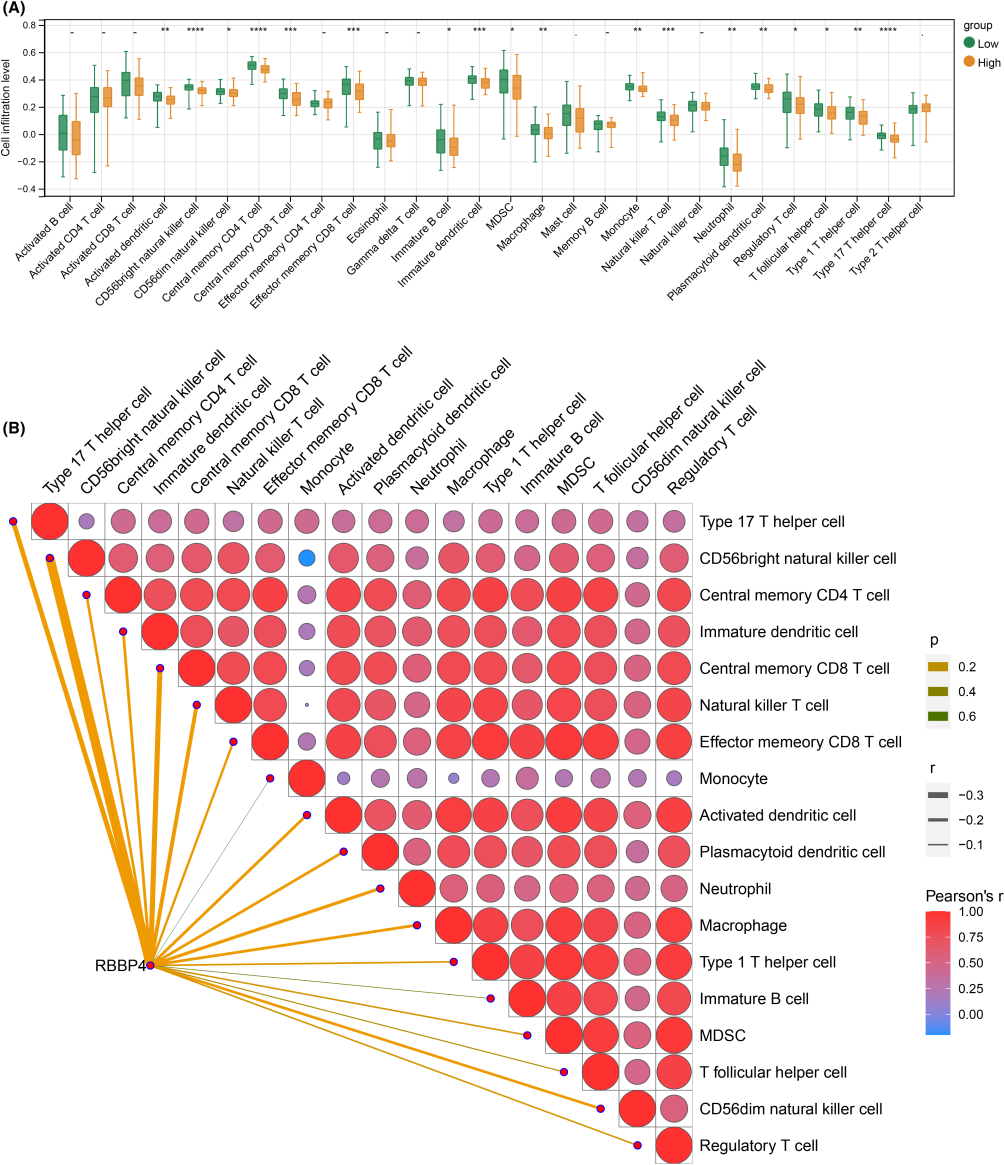
Associations between RBBP4 and immunomodulatory effects. (A) Infiltration levels of 28 types of immune cells in the high and low RBBP4 expression groups. (B) Correlations between RBBP4 expression and infiltration levels of 18 distinct immune cells. Line thickness denotes the absolute value of the correlation, with thicker lines representing stronger correlations and yellower line color indicating greater significance. *p < 0.05; **p < 0.01; ***p < 0.001; ****p < 0.0001; −, not significant.

We conducted ssGSEA to delve deeper into the possible link between RBBP4 expression and immune cell prevalence in NSCLC, quantifying the enrichment of diverse immune cell types in the samples. The analysis revealed a notable negative correlation of RBBP4 expression with 18 out of 28 evaluated immune cell types (*p* < 0.05) (Figure 4A), including type 17 T helper (Th17) cells, CD56‐bright natural killer (NK) cells, central memory CD4+ T (CD4+ TCM) cells, immature dendritic cells (imDCs), central memory CD8 T cells (CD8+ TCM) cells, NKT cells, effector memory CD8+ T cells, monocytes, activated dendritic cells, plasmacytoid dendritic cells (pDCs), neutrophils, macrophages, type 1 T helper (Th1) cells, immature B cells, myeloid‐derived suppressor cells (MDSCs), T follicular helper (Tfh) cells, CD56‐dim NK cells, and regulatory T cells (Tregs). Notably, the strongest negative correlation was observed for CD56‐bright NK cells, while the correlations with monocytes and immature B cells were relatively weak (Figure 4B). These data underscore the potential link between RBBP4 expression and the degree of immune cell infiltration in NSCLC.

### 3.5 RBBP4 expression in NSCLC is significantly correlated with autophagy‐related gene expression

**FIGURE 5:**
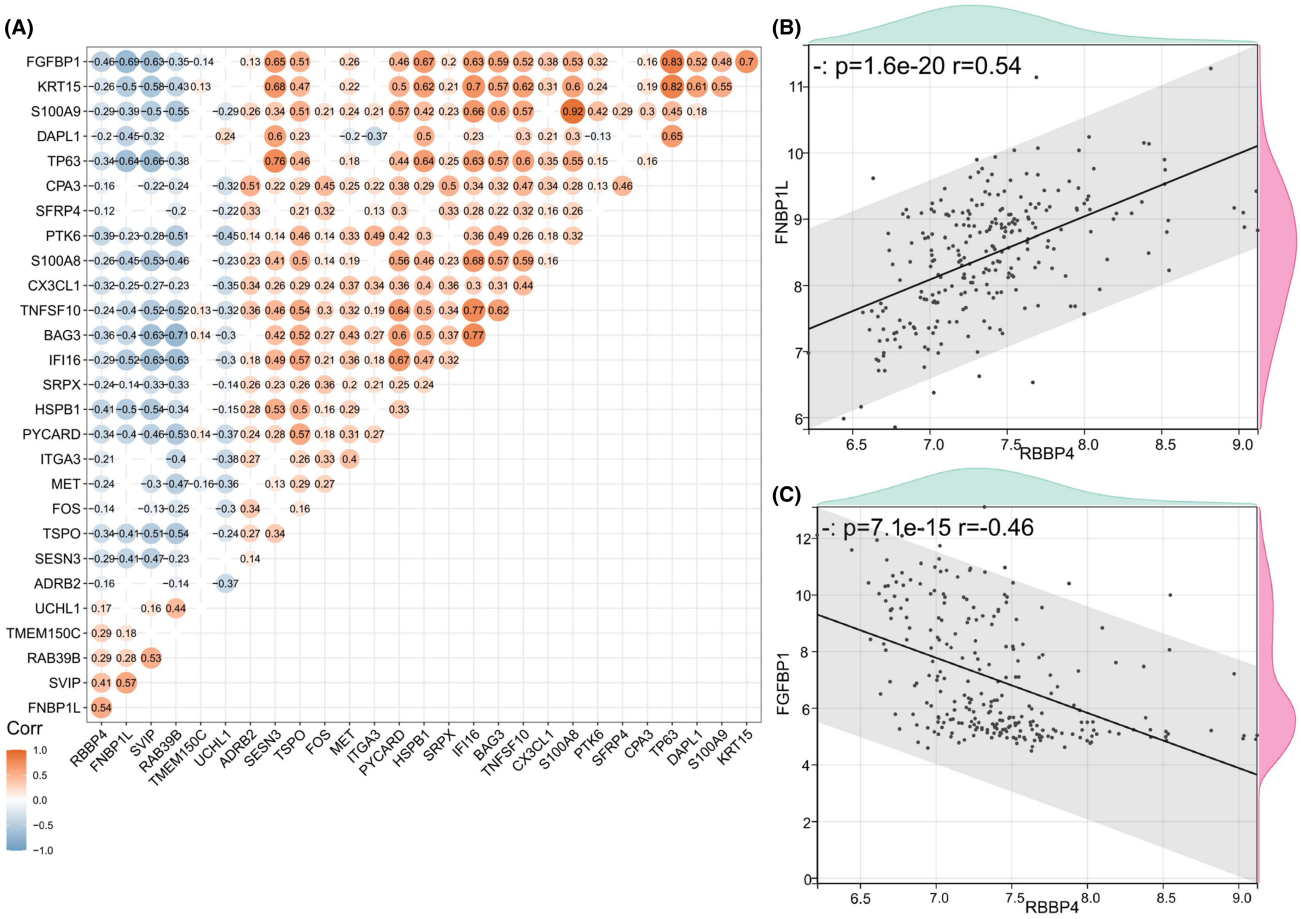
Correlation analysis of RBBP4 expression with autophagy genes. (A) Heatmap illustrating the correlations between RBBP4 gene expression and differential autophagy genes Blue indicates a negative correlation, red indicates a positive correlation, and blank indicates a nonsignificant correlation. Correlation coefficients were calculated using Pearson's method, with values ranging from −1 (strong negative correlation) to +1 (strong positive correlation). Significance was determined at *p* < 0.05. (B) Correlation analysis of RBBP4 and FNBP1L expression. The right‐side density curve represents the FNBP1L distribution, while the upper curve represents the RBBP4 distribution. (C) Correlation analysis of RBBP4 and FGFBP1 expression. Density curves for FGFBP1 and RBBP4 are similar to those in Figure 5B.

Enriched pathways in the high‐expression group (Figure 3A) were closely associated with DNA replication and repair. Previous studies have suggested that DNA repair systems and autophagic responses are coactivated to combat DNA damage.^38^, ^39^ The cell cycle and autophagy exhibit mutual coordination and regulation.^40^ Therefore, we investigated whether RBBP4 is involved in autophagy regulation. We assessed the relationship between RBBP4 expression and autophagy‐related genes in NSCLC by analyzing gene expression differences in the GSE30219 dataset between groups with high and low RBBP4 levels. This comparison yielded 571 differentially expressed genes (DEGs‐GSE30219) that were significantly correlated with RBBP4 expression (Supplementary Table S2). Comparison of these genes with known autophagy‐related genes revealed 27 autophagy‐related differentially expressed genes (AR‐DEGs‐GSE30219) (Table 1). Correlation analysis between RBBP4 and AR‐DEGs‐GSE30219 demonstrated significant associations (Figure 5A). Specifically, positive correlations were observed for genes including FNBP1L, SVIP, RAB39B, TMEM150C, and UCHL1, with FNBP1L exhibiting the strongest positive correlation (Figure 5B). Conversely, other genes demonstrated negative correlations, with FGFBP1 having the strongest association (Figure 5C).

**TABLE 1:** Autophagy‐associated differentially expressed genes in the GSE30219 dataset.

Gene	logFC	AveExpr	*t*	*p*‐Value	*p*.adjust value	*B*
FNBP1L	0.764252683	8.447605158	6.432153724	6.17E‐10	6.58E‐08	12.28051504
SVIP	0.678116714	7.246573296	4.590063603	6.96E‐06	0.000126406	3.401602339
RAB39B	0.62543392	4.345798826	3.387424631	0.000817041	0.005400507	−1.028829543
TMEM150C	0.625387756	6.382082955	4.121147512	5.10E‐05	0.000617613	1.534797886
UCHL1	0.593755489	6.815881075	3.189157135	0.001605263	0.009076518	−1.64350899
ADRB2	−0.593014067	6.120404464	−3.858348191	0.000144666	0.00140168	0.565291979
SESN3	−0.595203883	5.138776099	−4.197595886	3.73E‐05	0.00048003	1.827309683
TSPO	−0.605033555	9.995438703	−5.571935642	6.39E‐08	2.89E‐06	7.845412073
FOS	−0.616267008	9.249494018	−3.100778807	0.002146778	0.011411291	−1.906500523
MET	−0.620364223	7.158076303	−4.032463749	7.30E‐05	0.000823454	1.201356258
ITGA3	−0.639000045	6.995393957	−4.314665285	2.29E‐05	0.000328651	2.284269907
PYCARD	−0.642934969	8.291930921	−4.461981887	1.22E‐05	0.000200506	2.874627736
HSPB1	−0.649834779	11.79171423	−5.288810079	2.65E‐07	9.19E‐06	6.493028825
SRPX	−0.672610932	6.91844788	−4.134302203	4.83E‐05	0.000590349	1.58479769
IFI16	−0.693660148	9.19149953	−3.925508619	0.000111404	0.001140048	0.80770018
BAG3	−0.725359463	9.132360475	−4.786395505	2.88E‐06	6.23E‐05	4.233705234
TNFSF10	−0.728458723	9.409407995	−3.910286174	0.000118237	0.001197815	0.752431983
CX3CL1	−0.750149767	6.715132071	−5.588265664	5.88E‐08	2.70E‐06	7.925097932
S100A8	−0.781281289	7.577745076	−3.114437414	0.002053331	0.011026945	−1.866302329
PTK6	−0.78273107	5.694139395	−6.555654882	3.05E‐10	3.73E‐08	12.95522713
SFRP4	−0.844903537	6.799316157	−3.668499358	0.000296991	0.00246096	−0.09977564
CPA3	−0.889237163	7.617418515	−3.80865475	0.000175118	0.00162282	0.388319362
TP63	−0.918140734	5.261142408	−4.104344855	5.46E‐05	0.000654023	1.471134746
DAPL1	−0.935654919	6.958683465	−2.499189915	0.013077189	0.045243887	−3.512469465
S100A9	−1.024490759	9.63142573	−3.545597086	0.000465925	0.003502252	−0.514249752
KRT15	−1.261438935	7.508020507	−3.244315754	0.001334589	0.007844888	−1.475919381
FGFBP1	−1.676197582	7.019035396	−5.855622366	1.46E‐08	8.72E‐07	9.255193768

Abbreviations: AveExpr, average expression level; B, Bayes factor; logFC, log‐transformed fold change; *t*, *t*‐test statistic.

### 3.6 Analyzing the transcriptome and functionally enriching the profile of genes differentially expressed in PC9 cells with RBBP4 knockdown

**FIGURE 6:**
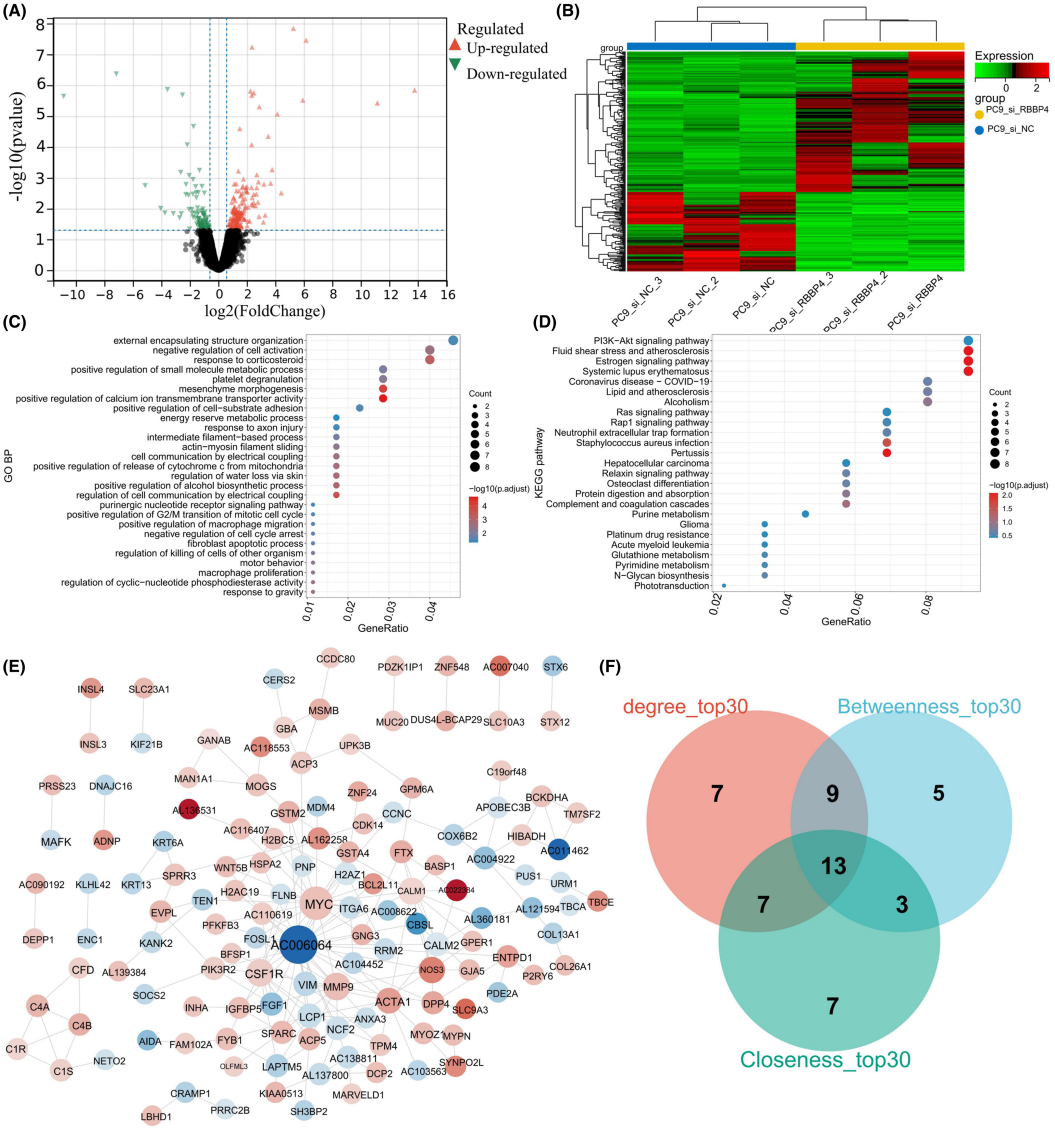
Transcriptomic profiling and functional pathway analysis in RBBP4‐knockdown PC9 Cells. (A) Volcano plot of DEGs between the si‐RBBP4 and si‐NC groups. Red dots represent upregulated genes screened based on |logFC| > 0.585 and *p* < 0.05, while green dots represent downregulated genes selected using the same criteria. Black dots indicate genes with no significant difference in expression. Gray dots represent genes with insufficient signal intensities for detection. (B) Heatmap of DEGs between the si‐RBBP4 and si‐NC groups. Red denotes upregulated gene expression, green indicates downregulated gene expression, and black signifies no significant change in gene expression. (C) GO enrichment analysis of DEGs‐PC9. (D) KEGG enrichment analysis of DEGs‐PC9. (E) PPI network of DEGs‐PC9. Red represents upregulated genes, blue represents downregulated genes, and darker colors indicate larger differences in expression. Larger nodes correspond to greater connectivity, while gray lines signify interactions between the proteins corresponding to the genes. (F) Venn diagram displaying the top 30 genes identified with the degree, betweenness, and closeness algorithms.

In our comparative analysis of the RBBP4 interference group (si‐RBBP4) and control group (si‐NC) of PC9 cell lines, we identified 174 upregulated and 98 downregulated genes (Figure 6A,B). Following this, we carried out both GO BP and KEGG pathway enrichment analyses on the DEGs identified in RBBP4 knockdown PC9 cells (DEGs‐PC9). GO analysis highlighted 326 biological processes that clustered into 27 distinct categories (Figure 6C). The DEGs‐PC9 were primarily associated with processes such as positive regulation of calcium ion transmembrane transporter activity and mesenchyme morphogenesis. KEGG pathway analysis identified 25 related pathways, with notable enrichment of fluid shear stress and atherosclerosis, estrogen signaling, systemic lupus erythematosus, and pertussis pathways (Figure 6D).

The resulting PPI network was composed of 207 reciprocal relationship pairs and 135 gene proteins (Figure 6E). The PPI network was imported into the CytoNCA plugin, where three distinct algorithms (for degree, betweenness, and closeness) were used to facilitate the identification of 135 DEGs‐PC9. After extracting the top 30 genes from each algorithm, we identified 13 overlapping genes as key DEGs‐PC9: AC006064, MYC, MMP9, CSF1R, CALM1, NOS3, ACTA1, VIM, CALM2, DPP4, GSTM2, RRM2, and AC138811 (Figure 6F). Notably, MYC is an inherently autophagy‐associated gene.

### 3.7 Screening of autophagy‐related differentially expressed genes associated with RBBP4

**FIGURE 7:**
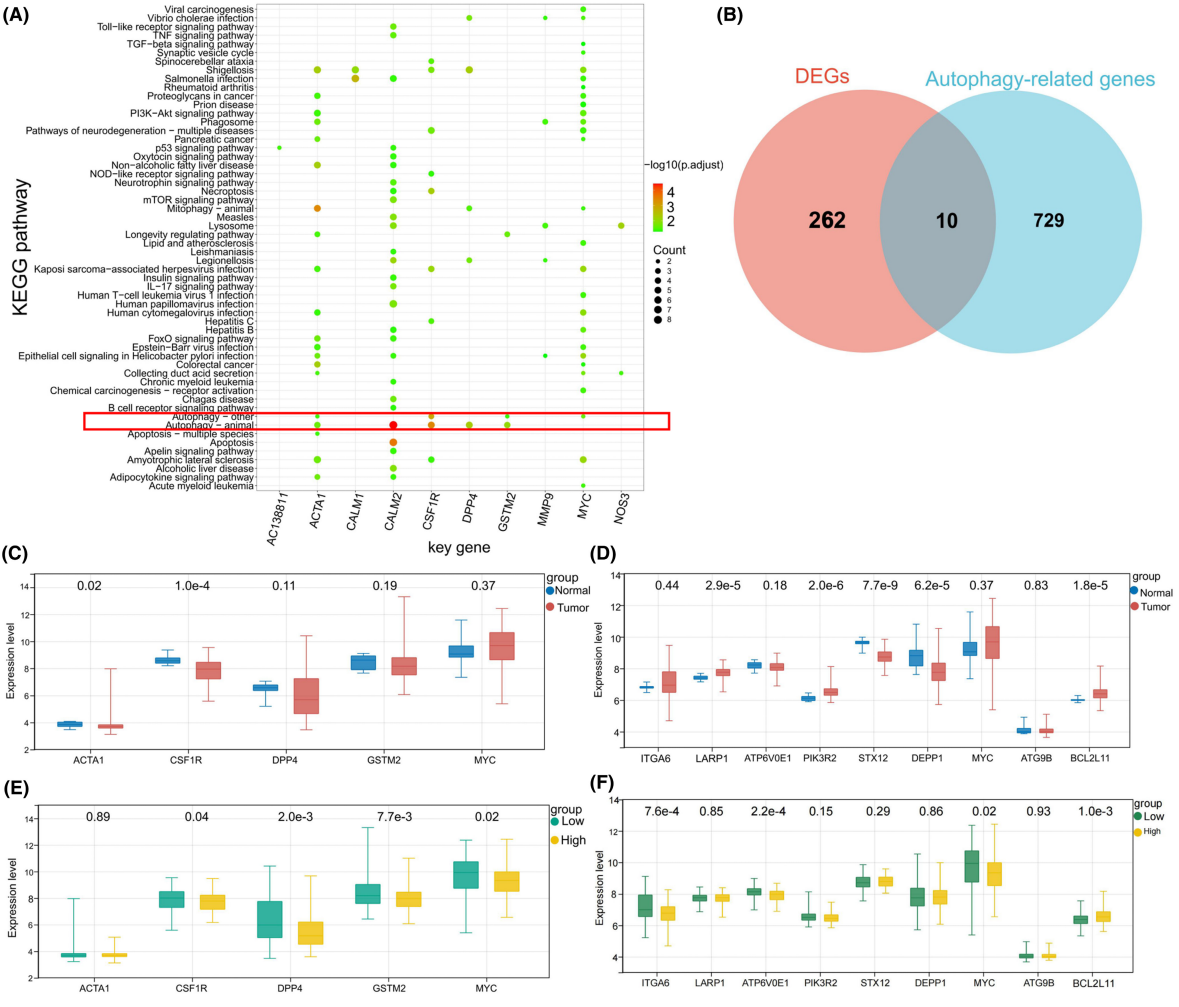
Selection and tumor expression of AR‐DEGs and their associations with RBBP4 expression. (A) KEGG enrichment analysis of each key DEG‐PC9. Redder bubble color indicates more significant enrichment. Larger bubble size indicates that more genes are enriched. (B) Venn diagram showing the overlap of DEGs‐PC9 and autophagy‐related genes. Among them, ACTA1, CALM2, CSF1R, DPP4, GSTM2, and MYC were enriched in autophagy‐related pathways. (C) Differential expression of five iAR‐DEGs between the tumor and normal groups in the GSE30219 dataset. (D) Differential expression of nine dAR‐DEGs between the tumor and normal groups in the GSE30219 dataset. (E) Differential expression of five iAR‐DEGs between the high and low RBBP4 expression groups in the GSE30219 dataset. (F) Differential expression of nine dAR‐DEGs between the high and low RBBP4 expression groups in the GSE30219 dataset.

To identify autophagy‐related differentially expressed genes between the si‐RBBP4 and si‐NC groups (AR‐DEGs‐PC9), we conducted Pearson correlation analysis between the 13 previously identified key DEGs‐PC9 and autophagy‐related genes sourced from the Human Autophagy Database (HADb) and related literature. Based on the established threshold value, 325 key relationship pairs of DEG‐PC9 and an autophagy gene were obtained (Supplementary Table S3). Subsequent KEGG pathway enrichment analysis revealed that CALM2, CSF1R, MYC, GSTM2, DPP4, and ACTA1 were enriched in the autophagy pathway (Figure 7A), leading us to classify these six genes as indirect autophagy‐related differentially expressed genes (iAR‐DEGs) associated with RBBP4.

For the identification of direct autophagy‐related differentially expressed genes (dAR‐DEGs) associated with RBBP4, we compared the list of DEGs‐PC9 with known autophagy genes (Figure 7B). Ten autophagy genes that overlapped with DEGs‐PC9 were identified: ITGA6, LARP1, ATP6V0E1, GBA, PIK3R2, STX12, DEPP1, MYC, ATG9B, and BCL2L11. In summary, we identified 15 RBBP4‐associated genes that are directly or indirectly related to autophagy in PC9 cells (MYC is common to iAR‐DEGs and dAR‐DEGs), underscoring the potential role of RBBP4 in the autophagy process.

### 3.8 AR‐DEGs associated with RBBP4 as predictive markers of NSCLC prognosis

To explore the roles of both iAR‐DEGs and dAR‐DEGs in the progression of NSCLC, we examined their expression disparities in tumor versus normal samples, and also comparing the distinct RBBP4 expression groups—high and low—in the GSE30219 dataset. Notably, among these 15 genes, iAR‐DEGs aligned with five in the GSE30219 dataset (ACTA1, CSF1R, DPP4, GSTM2, and MYC) while dAR‐DEGs aligned with nine genes in the GSE30219 dataset (ITGA6, LARP1, ATP6V0E1, PIK3R2, STX12, DEPP1, MYC, ATG9B, and BCL2L11). These results demonstrate that, compared to the normal group, LARP1, PIK3R2, and BCL2L11 were significantly overexpressed in NSCLC, while ACTA1, CSF1R, STX12, and DEPP1 were significantly underexpressed (*p* < 0.05) (Figure 7C,D). In addition, the results revealed significant negative correlations of RBBP4 with CSF1R, DPP4, GSTM2, MYC, ITGA6, and ATP6V0E1 in the GSE30219 dataset, while RBBP4 expression was significantly positively correlated with BCL2L11 expression (*p* < 0.05) (Figure 7E,F).

Next, we assessed the impact of iAR‐DEGs and dAR‐DEGs on RFS and DFS of NSCLC patients, and obtained nine results that exhibited pronounced differences. These results demonstrated that increased expression of MYC, LARP1, ATG9B, and BCL2L11, as well as decreased expression of DPP4, GSTM2, ATP6V0E1, and STX12 were significantly correlated with poorer RFS and DFS outcomes in NSCLC patients. However, the expression of CSF1R had no significant association with either RFS or DFS (Figures [Link], [Link]). The observed data imply that the expression levels of specific AR‐DEGs may serve as potential prognostic biomarkers in NSCLC, validating the predictive prognostic value of RBBP4.

### 3.9 Knockdown of RBBP4 induces autophagic cell death in PC9 cells

**FIGURE 8:**
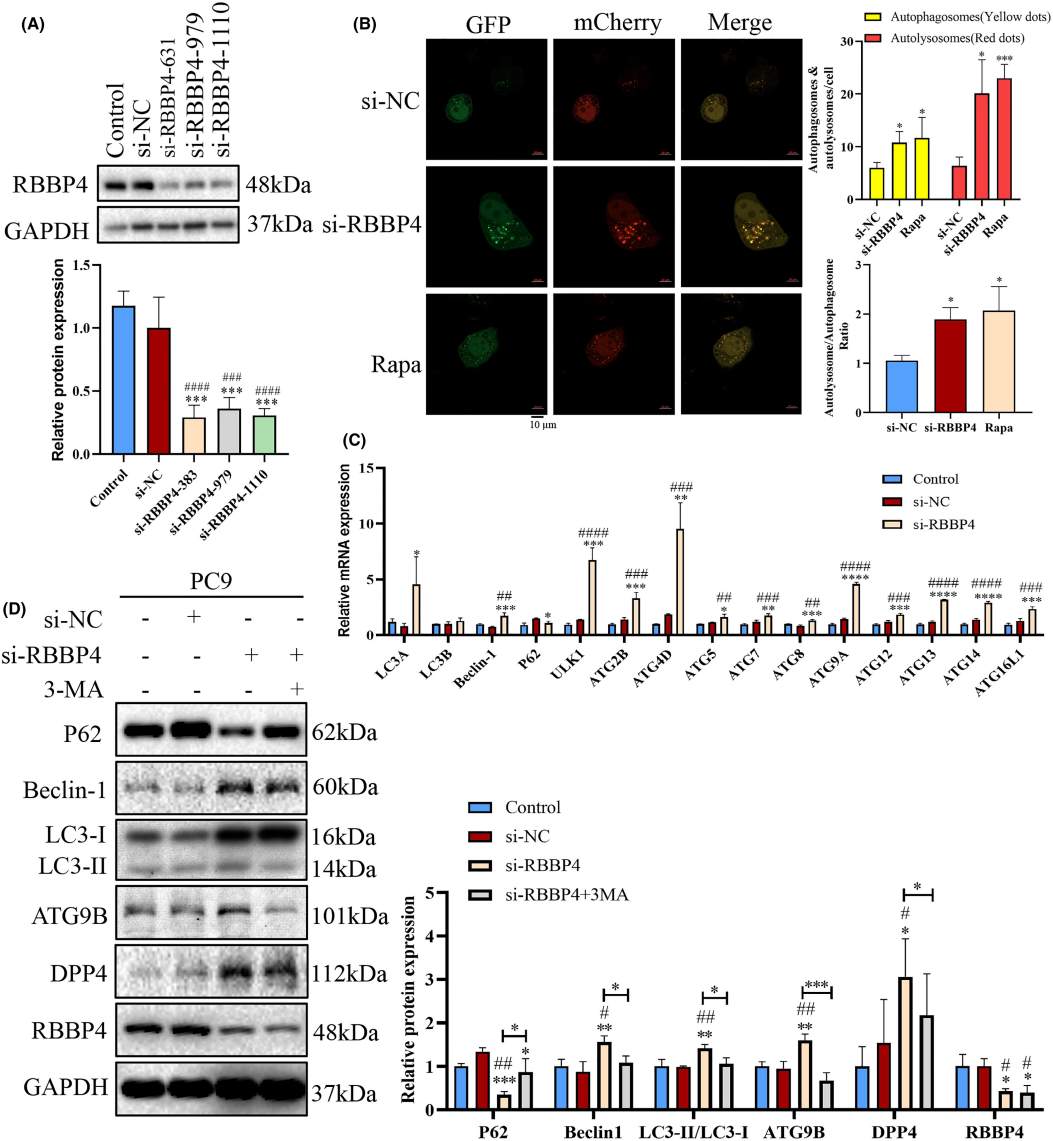
RBBP4 knockdown leads to enhanced autophagy in PC9 cells. (A) Suppression of RBBP4 expression in PC9 cells using si‐NC (negative control), si‐RBBP4‐631, si‐RBBP4‐979, and si‐RBBP4‐1110. (B) PC9 control cells or RBBP4 knockdown cells were transiently transfected with the mCherry‐GFP‐LC3B plasmid. Confocal microscopy was used to acquire images (left panel), and the average number of yellow puncta (autophagosomes) and red puncta (autolysosomes) per cell were quantified (top right). The ratio of autolysosomes to autophagosomes for each treatment group was calculated (bottom right). Rapamycin was used as a positive control. Scale bar = 10 μm, *p < 0.05, ***p < 0.001. (C) Real‐time PCR analysis of autophagy‐related gene expression in PC9 cells. White bars represent the control group, gray bars represent the si‐NC group, and black bars represent the si‐RBBP4 group. (D) Successful knockdown of RBBP4, followed by 24 h culturing and treatment with 0.25 mM 3‐MA. Western blotting was performed to assess the expression levels of P62, Beclin‐1, LC3B, DPP4, ATG9B, and RBBP4. GAPDH served as the internal reference for all proteins. Mean ± standard deviation (*n* = 3). #*p* < 0.05 versus the control group, ##*p* < 0.01 versus the control group, ###*p* < 0.001 versus the control group, ####*p* < 0.0001 versus the control group, *p < 0.05 versus the si‐NC group, **p < 0.01 versus the si‐NC group, ***p < 0.001 versus the si‐NC group, and ****p < 0.0001 versus the si‐NC group.

WB analysis confirmed the knockdown efficiency of three RBBP4 siRNA sequences. Each of the three siRNA sequences successfully suppressed RBBP4 expression in PC9 cells (Figure 8A). Among the sequences used, si‐RBBBP‐631 exhibited the most significant inhibitory effect and was subsequently employed for RBBP4 knockdown in further experiments, designated si‐RBBP4. Our initial investigations focused on RBBP4's potential involvement in autophagy regulation within PC9 cells. After RBBP4 knockdown, the mCherry‐GFP‐LC3B dual fluorescence system not only revealed an increased count of autophagosomes and autolysosomes within PC9 cells but also demonstrated a significant rise in the ratio of autolysosomes to autophagosomes (Figure 8B). This indicates an enhancement of autophagic flux in PC9 cells after RBBP4 knockdown, suggesting a potential inhibitory role of RBBP4 against autophagy. Real‐time quantitative PCR indicated that after RBBP4 knockdown, the mRNA expression levels of LC3A, Beclin‐1, ULK1, ATG2B, ATG5, ATG7, ATG8, ATG9A, ATG12, ATG13, ATG14, and ATG16L1 significantly increased, while LC3B showed a nonsignificant increasing trend, and P62 mRNA was markedly downregulated (Figure 8C). Furthermore, western blot analysis echoed these findings, showing elevated levels of LC3‐II and Beclin‐1, a higher LC3‐II/I ratio, and reduced P62. Remarkably, the application of the autophagy inhibitor 3‐MA reversed these expression patterns. Moreover, focusing on the two AR‐DEGs, DPP4 and ATG9B, we observed that RBBP4 knockdown increased the expression levels of DPP4 and ATG9B, and these changes were mitigated by treatment with 3‐MA (Figure 8D). These experimental findings conclusively demonstrate that RBBP4 functions as an autophagy inhibitor in PC9 cells, and its knockdown significantly enhances cellular autophagy.

**FIGURE 9:**
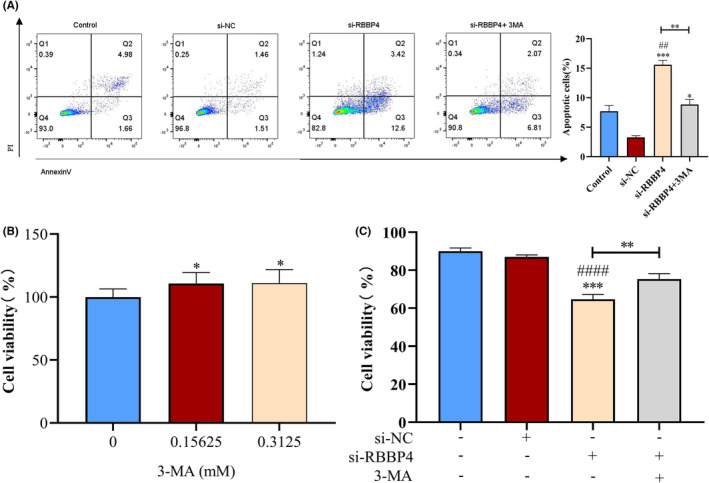
Knockdown of RBBP4 can enhance apoptosis and cell death in PC9 cells. (A) After successful knockdown of RBBP4, cells were cultured for 24 h and treated with 0.25 mM 3‐MA. Apoptosis in PC9 cells was assessed via comparison of annexin V staining among the conditions of si‐NC, si‐RBBP4, and si‐RBBP4 with 3‐MA treatment. (B) After successfully knocking down RBBP4 in PC9 cells, the cells were treated with varying concentrations of 3‐MA (0 mM, 0.15625 mM, and 0.3125 mM), cell viability was assessed using the CCK‐8 assay. (C) Evaluation of the viability ratio of PC9 cells treated with si‐NC and si‐RBBP4, with and without administration of 3‐MA (0.25 mM) to the si‐RBBP4‐treated group, as determined using a cell counter. Mean ± standard deviation (*n* = 3). **p < 0.01, ##*p* < 0.01 versus the control group, ####*p* < 0.0001 versus the control group, *p < 0.05 versus the si‐NC group, and ***p < 0.001 versus the si‐NC group.

To further clarify whether knockdown of RBBP4 leads to apoptosis in PC9 cells through enhanced autophagy, the function of RBBP4 in regulating cell apoptosis was examined. Flow cytometry analysis indicated a pronounced increase in the apoptosis rate following si‐RBBP4 treatment, particularly in the early period after treatment (Figure 9A). Notably, this apoptosis increase was significantly attenuated upon 3‐MA treatment. To delve deeper into whether RBBP4 knockdown‐induced autophagy might affect cell survival, we exposed si‐RBBP4‐treated PC9 cells to various concentrations of 3‐MA and assessed cell viability. Our findings reveal a significant increase in the viability of PC9 cells at 3‐MA concentrations of 0.15625 and 0.3125 mM compared to the RBBP4‐knockdown group (Figure 9B). Further, cells with RBBP4 knockdown were treated with 0.25 mM 3‐MA and stained with trypan blue, a dye selective for dead cells. Relative to the si‐NC control group, a pronounced decrease in viable cells was found in the si‐RBBP4 group; however, this trend was markedly reversed upon 3‐MA treatment (Figure 9C). This comprehensive analysis suggests that knockdown of RBBP4 induces autophagic cell death in PC9 cells, and this autophagic activity contributes to the growth inhibition observed with RBBP4 knockdown.

### 3.10 RBBP4 knockdown inhibits tumor growth and enhances apoptosis and autophagy in animal models

**FIGURE 10:**
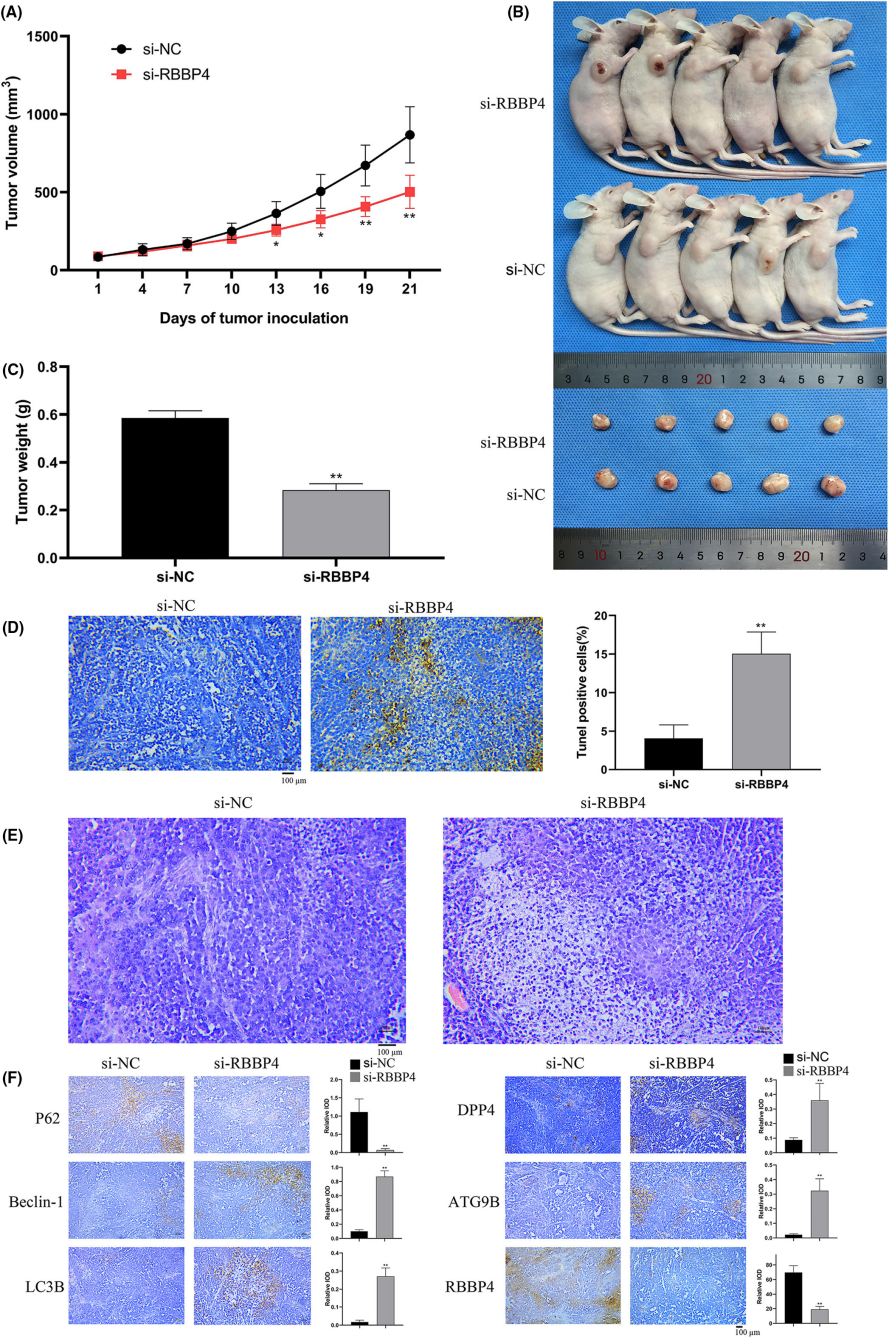
Effects of RBBP4 on the growth of PC9 cells in vitro. (A) Tumor volume was measured every 3 days during the experimental period (*n* = 5). (B and C) Tumors were surgically excised and weighed (*n* = 5). (D) The TUNEL assay was employed to detect apoptosis. Positive apoptotic nuclei appeared brown or brown‐black, and results are expressed as the percentage of total cells showing positive apoptotic staining at 200 × magnification. Compared to the si‐NC group, tissues treated with si‐RBBP4 showed a significant increase in TUNEL‐positive nuclei (*p* < 0.05). (E) Representative images of hematoxylin and eosin staining of tumors from both groups (magnification, 200×). (F) Immunohistochemistry was employed to determine the protein levels of P62, Beclin‐1, LC3B, DPP4, ATG9B, and RBBP4 in tumor tissues of the si‐NC and si‐RBBP4 groups. Scale bar = 100 μm, Mean ± standard deviation (*n* = 3). *p < 0.05; **p < 0.01; ***p < 0.001; and ****p < 0.0001.

Tumor growth was monitored every 3 days in both groups of mice, and the data were plotted to generate growth curves. Notably, 13 days after multiple siRNA injections, the si‐RBBP4 group exhibited significant suppression of tumor progression compared to the control group (*p* < 0.05) (Figure 10A). At day 21, the tumors were weighed; the average weight was 0.585 ± 0.0342 g in the si‐NC group and 0.28 ± 0.029 g in the si‐RBBP4 group, indicating a significant decrease in tumor weight for the si‐RBBP4 group relative to the si‐NC group (*p* < 0.01) (Figure 10B,C).

Tumor cell apoptosis was assessed using the TUNEL assay. Nuclei stained brown or brown‐black indicated apoptosis and were recorded as TUNEL‐positive nuclei. Compared to the si‐NC group, the si‐RBBP4 group exhibited a significant increase in the percentage of TUNEL‐positive nuclei (*p* < 0.05) (Figure 10D), indicating that si‐RBBP4 can increase the apoptosis rate of PC9 xenografts. Hematoxylin and eosin staining revealed that tumor cells in the si‐NC group exhibited robust growth with high microvascular density, as well as observable nuclear and cellular division. By contrast, the si‐RBBP4 group demonstrated a marked increase in tumor necrosis, accompanied by pronounced nuclear features of condensation and fragmentation (Figure 10E). These findings suggest that RBBP4 knockdown significantly enhances cellular apoptosis and necrosis in the PC9 xenograft tumor model. Subsequently, we employed immunohistochemistry to assess the expression of autophagy‐related proteins in both groups. Compared to the si‐NC group, the si‐RBBP4 group exhibited significant upregulation of Beclin‐1 and LC3B expression and notable downregulation of P62 expression. Moreover, we further validated the expression of AR‐DEGs, and found that compared to the si‐NC group, the si‐RBBP4 group exhibited marked elevation of DPP4 and ATG9B expression (Figure 10F), suggesting that knockdown of si‐RBBP4 promotes cellular autophagy. Taken together, these findings suggest that RBBP4 knockdown may inhibit tumor growth and augment tumor cell apoptosis through enhancement of lethal autophagy.

## 4 DISCUSSION

We observed elevated RBBP4 expression in NSCLC tissues, in accordance with prior findings of its upregulation in NSCLC tumors,^41^, ^42^ which suggests that RBBP4 has a potential oncogenic role in lung cancer. RBBP4 expression appeared to increase with advancing NSCLC stage, as observed with the transitions from N0 to N3 and from M1 to M0. In a previous study, higher RBBP4 levels were noted in acute leukemia and blast crisis chronic myeloid leukemia than in non‐leukemic states and the chronic phase of chronic myeloid leukemia,^43^ suggesting an association with malignancy progression. Among NSCLC subtypes, RBBP4 was notably upregulated in ADC versus SQC. Similarly, Lohavanichbutr et al.^44^ reported differential RBBP4 expression between human papilloma virus (HPV)‐positive and HPV‐negative oropharyngeal cancers. These findings suggest that RBBP4 plays diverse roles across NSCLC subtypes, emphasizing the need to consider the specific subtype before pursuing RBBP4‐targeted therapies.

The Kaplan–Meier survival analysis indicated poorer RFS and DFS in the high‐expression group relative to those with low expression. Notably, elevated RBBP4 levels are associated with poor prognosis in various tumors, including GBM,^45^ acute myeloid leukemia,^16^ and colorectal cancer.^24^ This association suggests that RBBP4 could be an essential indicator for prognosis in NSCLC, indicating potential clinical significance.

The tumor microenvironment (TME) encompasses the extracellular matrix, cancer‐associated fibroblasts, vascular cells, and infiltrating immune cells.^46^, ^47^, ^48^ Recently, immune cell infiltration of solid tumors has emerged as a key factor in TME‐related carcinogenesis.^49^ The presence of immune cells within tumor tissues correlates with lymph node metastasis and the prognosis in lung adenocarcinoma.^50^, ^51^ Our study identified an inverse relationship between RBBP4 levels and the infiltration levels of 18 different immune cell types, with CD56‐bright NK cells showing the most pronounced association. Previous research has shown that CD56‐bright NK cells are notably enriched in NSCLC tissues, where they function predominantly as cytokine producers and possess antitumor capabilities.^52^, ^53^ We speculate that high RBBP4 expression in NSCLC might inhibit antitumor immune responses, leading to tumor immune evasion and hastening tumor progression. The adverse prognosis of NSCLC patients with increased RBBP4 may be due to a diminished overall immune response. However, further experiments are essential to validate this possibility.

GSEA revealed that pathways associated with genomic instability are predominantly enriched in the high‐expression group, potentially accounting for the observed adverse prognosis. RBBP4, acting as a histone chaperone,^54^ is involved in DNA damage repair and chromatin remodeling.^55^, ^56^ Changes in its expression can induce DNA damage.^57^ RBBP4 plays a pivotal role in the regulation of the cell cycle: As a core component of the MuvB complex, it is involved in the assembly of the DREAM complex by binding to p130/E2F4/DP1 during the G0 phase, thereby inhibiting cell cycle progression.^58^ In the G2/S phase, the MuvB complex containing RBBP4 can interact with activated transcription factors B‐MYB and forkhead box M1 (FOXM1) to form the MMB complex, further modulating the cell cycle.^59^ Additionally, RBBP4 is a member of the RB protein family. It interacts with RB, thereby inhibiting cell cycle progression and cellular growth.^6^

Our study revealed a significant association between RBBP4 and various genes involved in autophagy, including FNBP1L and FGFBP1. FNBP1L, which interacts with ATG3, is essential in restricting intracellular bacterial proliferation.^60^ FGFBP1 has deleterious effects in various cancers.^61^, ^62^ However, the specific roles of RBBP4 in tumor autophagy in relation to these two genes require further investigation.

Autophagic cell death can be defined as instances in which cell death is inhibited when autophagy is suppressed using chemical inhibitors or genetic means.^63^ Cancer cell eradication may occur via autophagic cell death mechanisms. Studies have indicated that diminished autophagic processes are linked to tumor development, for example, low Beclin‐1 levels are observed in ovarian cancer, breast cancer, and prostate cancer.^64^ Typically, increased LC3‐II and reduced P62 expression are considered biomarkers of autophagy induction, while Beclin‐1 expression often increases with autophagy.^65^ Our tests showed that RBBP4 knockdown resulted in increased levels of LC3‐II and Beclin‐1, along with a reduction in P62 levels, thereby confirming RBBP4's role in inhibiting autophagy in NSCLC. Flow cytometry indicated that the knockdown of RBBP4 promotes early apoptosis in PC9 cells, and that this effect is inhibited by 3‐MA. Furthermore, 3‐MA suppresses the induction of cell death with RBBP4 knockdown. These findings collectively suggest that the downregulation of RBBP4 leads to autophagic cell death in NSCLC.

In addition, we assessed DPP4 and ATG9B protein expression, revealing that RBBP4 knockdown significantly increased DPP4 and ATG9B expression. DPP4 may indirectly affect autophagy through its influence on immune cell activity.^66^ Although we verified the association between RBBP4 and immune cell infiltration, a direct link between RBBP4 and DPP4 remains elusive. ATG9B, belonging to the autophagy‐related protein group, is crucial for the formation and maturation of autophagosomes.^67^, ^68^ Therefore, knockdown or deletion of RBBP4 may affect the expression of other proteins, such as DPP4 and ATG9B, which may in turn affect autophagy and immune cell functions.

In summary, RBBP4 is significantly overexpressed in NSCLC, suggesting its potential as a biomarker for NSCLC. High expression of RBBP4 may be associated with poor prognosis in NSCLC patients. Knockdown of RBBP4 induces autophagy‐mediated cell death in NSCLC cells both in vivo and in vitro, laying the groundwork for a deeper understanding of the anticancer mechanisms of RBBP4.

## AUTHOR CONTRIBUTIONS

**Yajing Zhan:** Data curation (equal); formal analysis (equal); investigation (equal); methodology (equal); validation (equal); visualization (equal); writing – original draft (lead); writing – review and editing (equal). **Zhiqian Zhang:** Data curation (equal); formal analysis (equal); investigation (equal); methodology (equal); writing – review and editing (equal). **Ankang Yin:** Investigation (equal); software (equal); visualization (equal); writing – review and editing (equal). **Xiyang Su:** Software (equal); visualization (equal); writing – review and editing (equal). **Nan Tang:** Data curation (equal); investigation (equal); validation (equal); writing – review and editing (equal). **Yi Chen:** Writing – review and editing (equal). **Zebin Zhang:** Writing – review and editing (equal). **Wei Chen:** Funding acquisition (equal); project administration (equal); resources (equal); writing – review and editing (equal). **Juan Wang:** Conceptualization (equal); data curation (equal); funding acquisition (equal); methodology (equal); project administration (equal); resources (equal); supervision (equal); writing – review and editing (equal). **Wei Wang:** Conceptualization (equal); funding acquisition (equal); project administration (equal); resources (equal); supervision (equal); writing – review and editing (equal).

## FUNDING INFORMATION

This work was supported by the National Natural Science Foundation of China (Grant No. 82004007 and 81,774,026).

## CONFLICT OF INTEREST STATEMENT

The authors have no conflict of interest.

## ETHICS STATEMENT

All animal experiments were reviewed and approved by the Animal Experimentation Ethics Committee of Tongde Hospital of Zhejiang Province and conducted in accordance with the approved guidelines.

## Supporting information


Figure S1.

Figure S2.

Table S1.

Table S2.

Table S3.

## Data Availability

The original contributions presented in this study are included in the article itself and/or Supplementary Material. The corresponding authors can be contacted with any reasonable inquiries.
