# Supplementary figures and images for: RBBP4: A novel diagnostic and prognostic biomarker for non‐small‐cell lung cancer correlated with autophagic cell death
Source: Cancer Med. 2024 Aug 7;13(15):e70090. doi: 10.1002/cam4.70090 (PMC11304277; doi:10.1002/cam4.70090)

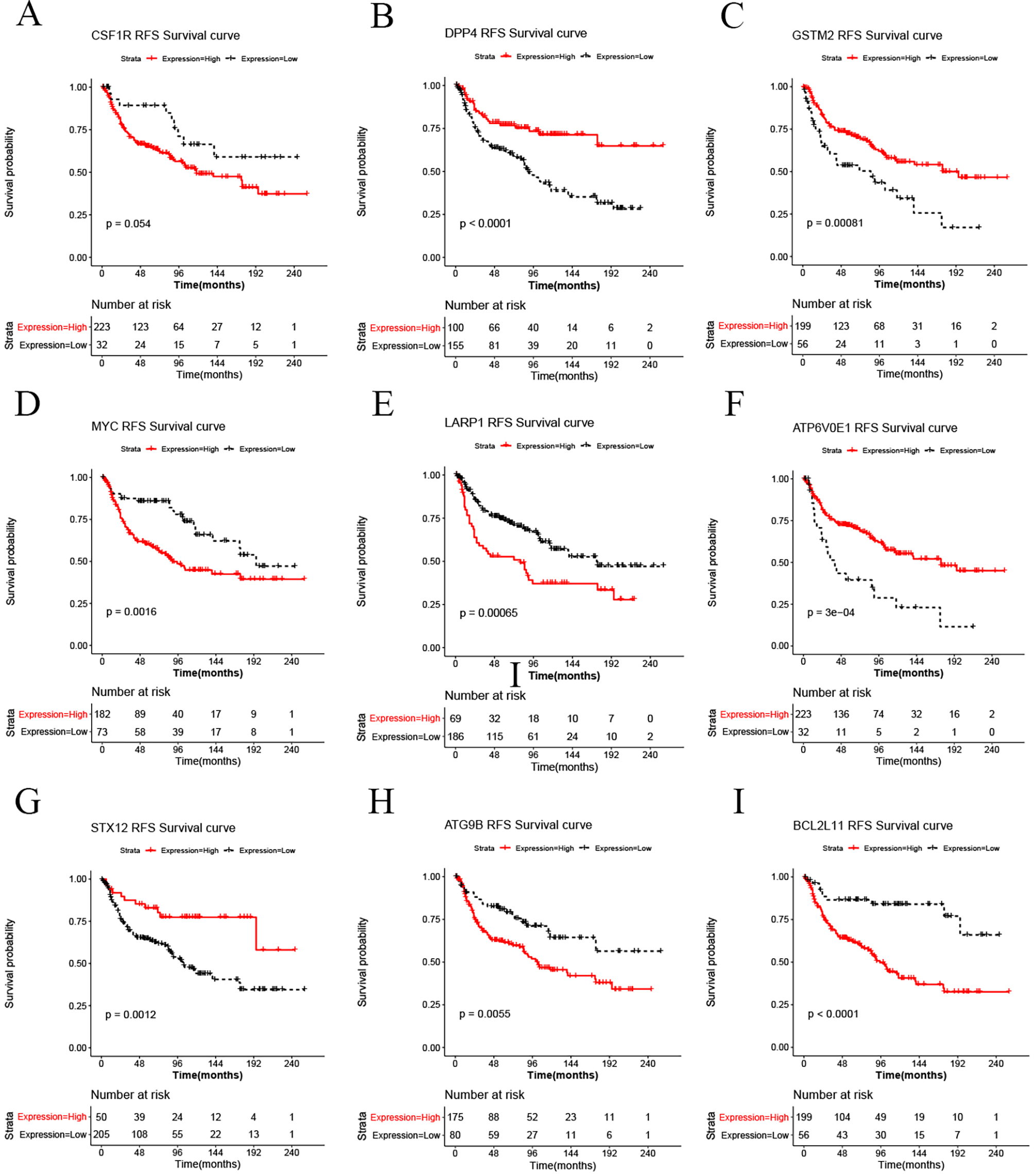

Supplement: Supplementary file 1 — Figure S1. [file CAM4-13-e70090-s001.tif]

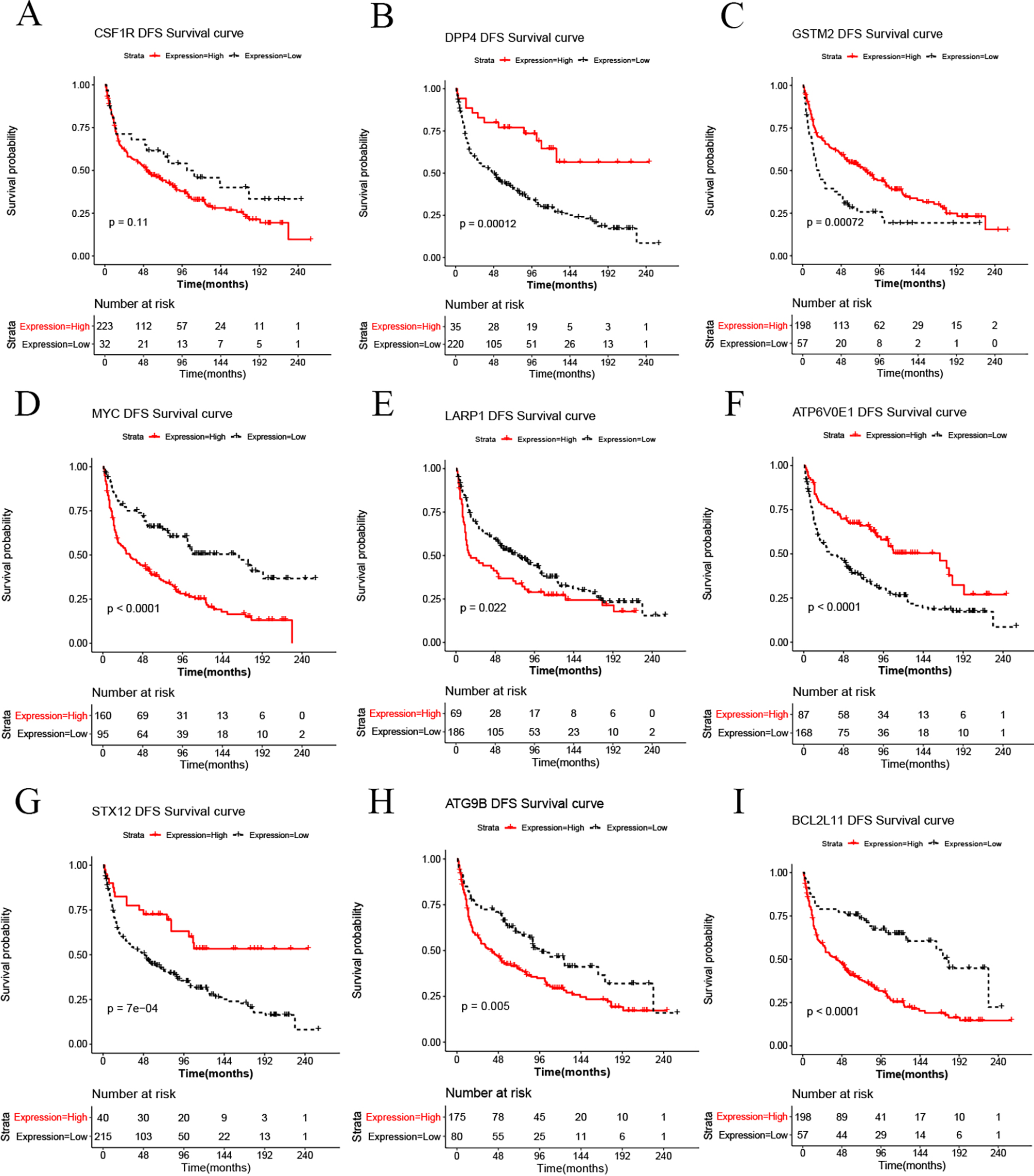

Supplement: Supplementary file 2 — Figure S2. [file CAM4-13-e70090-s003.tif]
