# Supplementary material for: RBBP4: A novel diagnostic and prognostic biomarker for non‐small‐cell lung cancer correlated with autophagic cell death
Source: Cancer Med. 2024 Aug 7;13(15):e70090. doi: 10.1002/cam4.70090 (PMC11304277; doi:10.1002/cam4.70090)
Supplement: Supplementary file 3 — Table S1. [file CAM4-13-e70090-s005.docx]

| Table S1 Primer sequences for real-time fluorescent quantitative PCR | | |
| --- | --- | --- |
| Primer name | **Sequence** (5'--3') | |
|  | Forward primer | Reverse primer |
| LC3A | AACATGAGCGAGTTGGTCAAG | GCTCGTAGATGTCCGCGAT |
| LC3B | GATGTCCGACTTATTCGAGAGC | TTGAGCTGTAAGCGCCTTCTA |
| Beclin-1 | CCATGCAGGTGAGCTTCGT | GAATCTGCGAGAGACACCATC |
| P62 | GCACCCCAATGTGATCTGC | CGCTACACAAGTCGTAGTCTGG |
| ULK1 | GGCAAGTTCGAGTTCTCCCG | CGACCTCCAAATCGTGCTTCT |
| ATG2B | AACTGCTGACGAATCCTCAGG | GGGGTTCCAGCTAGGTGAGA |
| ATG4D | GGAACAACGTCAAGTACGGTT | CTCGCCCTCGAAACGGTAG |
| ATG5 | AAAGATGTGCTTCGAGATGTGT | CACTTTGTCAGTTACCAACGTCA |
| ATG7 | CAGTTTGCCCCTTTTAGTAGTGC | CCAGCCGATACTCGTTCAGC |
| ATG8 | ACTCGCTGGAACACAGATGC | TCTGAGAGCCTGAGACCTTTT |
| ATG9A | CTGCCCTTCCGTATTGCAC | CTCACGTTTGTGGATGCAGAT |
| ATG12 | CTGCTGGCGACACCAAGAAA | CGTGTTCGCTCTACTGCCC |
| ATG13 | TTGCTATAACTAGGGTGACACCA | CCCAACACGAACTGTCTGGA |
| ATG14 | GCGCCAAATGCGTTCAGAG | AGTCGGCTTAACCTTTCCTTCT |
| ATG16L1 | AACGCTGTGCAGTTCAGTCC | AGCTGCTAAGAGGTAAGATCCA |
| GAPDH | GGAGCGAGATCCCTCCAAAAT | GGCTGTTGTCATACTTCTCATGG |
