# Supplementary material for: RBBP4: A novel diagnostic and prognostic biomarker for non‐small‐cell lung cancer correlated with autophagic cell death
Source: Cancer Med. 2024 Aug 7;13(15):e70090. doi: 10.1002/cam4.70090 (PMC11304277; doi:10.1002/cam4.70090)
Supplement: Supplementary file 4 — Table S2. [file CAM4-13-e70090-s004.docx]

| Table S2 571 DEGs associated with RBBP4 in the GSE30219 dataset. | | | | |
| --- | --- | --- | --- | --- |
| Gene | logFC | t | P.Value | adj.P.Val |
| HEPACAM2 | 2.448117582 | 7.75996 | 2.05E-13 | 9.84E-11 |
| ASCL1 | 2.150108627 | 6.44531 | 5.73E-10 | 6.14E-08 |
| TUBB2B | 1.850997415 | 5.93616 | 9.49E-09 | 6.27E-07 |
| PROM1 | 1.747774492 | 5.30071 | 2.50E-07 | 8.77E-06 |
| PCSK1 | 1.559646483 | 3.58629 | 0.0004019 | 0.0031188 |
| CLGN | 1.462081188 | 5.5641 | 6.65E-08 | 2.99E-06 |
| RNF182 | 1.456484348 | 7.20515 | 6.53E-12 | 1.65E-09 |
| MAGEA6 | 1.45603241 | 3.20061 | 0.0015452 | 0.0088074 |
| DDC | 1.444123289 | 4.98523 | 1.14E-06 | 2.98E-05 |
| CALCA | 1.394635186 | 4.92851 | 1.49E-06 | 3.68E-05 |
| INSM1 | 1.35294714 | 5.39535 | 1.56E-07 | 6.00E-06 |
| TFF3 | 1.334316007 | 4.3374 | 2.08E-05 | 0.0003036 |
| PPM1E | 1.302271229 | 7.98048 | 4.96E-14 | 3.03E-11 |
| SYT4 | 1.283134497 | 3.96336 | 9.60E-05 | 0.0010195 |
| SCG3 | 1.274654879 | 3.51501 | 0.0005202 | 0.003807 |
| MAGEA12 | 1.199910446 | 3.74762 | 0.0002208 | 0.0019357 |
| KIT | 1.196421123 | 5.01993 | 9.71E-07 | 2.62E-05 |
| ISL1 | 1.183241985 | 5.24549 | 3.28E-07 | 1.10E-05 |
| SOX11 | 1.170488333 | 5.84797 | 1.52E-08 | 9.00E-07 |
| SEC11C | 1.154302131 | 6.9048 | 3.98E-11 | 6.98E-09 |
| IGFBPL1 | 1.151147802 | 6.60072 | 2.36E-10 | 3.01E-08 |
| CCNE2 | 1.1364312 | 5.96681 | 8.05E-09 | 5.52E-07 |
| CDH2 | 1.133917195 | 6.73219 | 1.10E-10 | 1.65E-08 |
| ST8SIA6-AS1 | 1.117959094 | 5.64294 | 4.44E-08 | 2.18E-06 |
| TOX3 | 1.113847214 | 4.22574 | 3.32E-05 | 0.0004405 |
| CDC7 | 1.097761307 | 7.21857 | 6.02E-12 | 1.59E-09 |
| SH3GL2 | 1.096264603 | 4.08986 | 5.79E-05 | 0.0006819 |
| TSPYL5 | 1.039005214 | 4.72323 | 3.84E-06 | 7.92E-05 |
| IGFBP2 | 1.034941368 | 4.31411 | 2.29E-05 | 0.0003287 |
| FOXO6 | 1.031354776 | 7.31454 | 3.34E-12 | 9.77E-10 |
| DRAIC | 1.025147613 | 3.56651 | 0.0004319 | 0.0032978 |
| SPIN4 | 1.016961036 | 6.22792 | 1.94E-09 | 1.73E-07 |
| NPTX1 | 0.999259145 | 4.82007 | 2.47E-06 | 5.51E-05 |
| NUF2 | 0.994793291 | 4.3877 | 1.68E-05 | 0.0002559 |
| GNG4 | 0.9881524 | 4.70284 | 4.21E-06 | 8.44E-05 |
| FAM3B | 0.984903632 | 3.50995 | 0.0005297 | 0.0038483 |
| GRP | 0.979182493 | 3.58826 | 0.000399 | 0.003106 |
| WNK3 | 0.976851511 | 7.7441 | 2.27E-13 | 1.03E-10 |
| MNS1 | 0.96935849 | 7.4586 | 1.37E-12 | 4.46E-10 |
| MARCKSL1 | 0.966409932 | 9.04384 | 3.94E-17 | 7.22E-14 |
| UBE2QL1 | 0.966228194 | 3.75224 | 0.000217 | 0.0019122 |
| SCGN | 0.963988782 | 3.07397 | 0.0023417 | 0.0121893 |
| Table S2 continued | | | | |
| Gene | logFC | t | P.Value | adj.P.Val |
| EYA2 | 0.960862114 | 4.41136 | 1.52E-05 | 0.0002363 |
| ASRGL1 | 0.955430697 | 5.51091 | 8.72E-08 | 3.68E-06 |
| PROX1 | 0.95529998 | 5.08693 | 7.06E-07 | 2.04E-05 |
| CLDN3 | 0.942665288 | 3.86702 | 0.0001399 | 0.0013691 |
| MAP7D2 | 0.940091065 | 3.77862 | 0.0001964 | 0.0017681 |
| PTBP2 | 0.939046718 | 9.34035 | 4.97E-18 | 1.67E-14 |
| TMEM170B | 0.938680439 | 7.01098 | 2.11E-11 | 4.12E-09 |
| CDC20 | 0.936977445 | 4.04161 | 7.03E-05 | 0.0007996 |
| NOL4 | 0.931910466 | 4.44765 | 1.30E-05 | 0.0002095 |
| SLC36A4 | 0.930272008 | 8.49424 | 1.67E-15 | 1.78E-12 |
| FGL1 | 0.930170787 | 3.21251 | 0.001485 | 0.0085123 |
| SCGB2A1 | 0.929338261 | 3.51488 | 0.0005204 | 0.003807 |
| ZNF711 | 0.924229055 | 5.47518 | 1.05E-07 | 4.25E-06 |
| MLLT11 | 0.920645446 | 3.93139 | 0.0001089 | 0.0011206 |
| NDC80 | 0.920189668 | 4.26002 | 2.88E-05 | 0.0003938 |
| KIF2C | 0.919307596 | 5.42255 | 1.36E-07 | 5.39E-06 |
| NCAPG | 0.911761702 | 5.09217 | 6.88E-07 | 2.00E-05 |
| CPE | 0.904561143 | 3.85475 | 0.0001467 | 0.0014136 |
| IL17RB | 0.896071691 | 4.21688 | 3.44E-05 | 0.0004536 |
| RBBP4 | 0.887884778 | 19.1082 | 4.00E-51 | 8.06E-47 |
| DEPDC1 | 0.870200415 | 4.75027 | 3.39E-06 | 7.13E-05 |
| C14orf132 | 0.869906315 | 6.4535 | 5.47E-10 | 5.89E-08 |
| PKIA | 0.868398678 | 5.16931 | 4.75E-07 | 1.47E-05 |
| SNAP25 | 0.868314482 | 3.85226 | 0.0001481 | 0.0014245 |
| INA | 0.867415526 | 3.06024 | 0.0024477 | 0.0126237 |
| NEUROD1 | 0.866132549 | 5.39913 | 1.53E-07 | 5.93E-06 |
| NKX2-2 | 0.864967227 | 4.45602 | 1.25E-05 | 0.0002039 |
| SLC10A4 | 0.86458379 | 6.16583 | 2.73E-09 | 2.27E-07 |
| EZH2 | 0.861476996 | 4.4842 | 1.11E-05 | 0.0001863 |
| CHRNA5 | 0.86000532 | 4.44605 | 1.31E-05 | 0.0002106 |
| CTPS1 | 0.856441643 | 6.63935 | 1.89E-10 | 2.47E-08 |
| PSRC1 | 0.855193274 | 6.639 | 1.89E-10 | 2.47E-08 |
| ELOVL2 | 0.854391676 | 6.48776 | 4.50E-10 | 4.96E-08 |
| SCG5 | 0.853485454 | 2.71454 | 0.0070897 | 0.0285684 |
| ADCYAP1 | 0.853003118 | 4.26195 | 2.85E-05 | 0.0003912 |
| RNF183 | 0.852306992 | 4.88049 | 1.87E-06 | 4.41E-05 |
| KIF5C | 0.841209422 | 5.63943 | 4.52E-08 | 2.21E-06 |
| HES6 | 0.838861366 | 8.37197 | 3.79E-15 | 3.32E-12 |
| KLHL23 | 0.828826562 | 7.55493 | 7.50E-13 | 2.65E-10 |
| KIF4A | 0.828756489 | 4.29707 | 2.46E-05 | 0.0003469 |
| NEK2 | 0.827762053 | 4.13619 | 4.80E-05 | 0.0005869 |
| LINC01419 | 0.824122686 | 4.41048 | 1.52E-05 | 0.0002368 |
| Table S2 continued | | | | |
| Gene | logFC | t | P.Value | adj.P.Val |
| RBM38 | 0.820720787 | 7.01676 | 2.04E-11 | 4.12E-09 |
| TMEM178A | 0.81921479 | 3.69949 | 0.0002646 | 0.0022432 |
| LIN28B | 0.818998852 | 4.18749 | 3.89E-05 | 0.0004963 |
| RALYL | 0.817007265 | 4.65343 | 5.25E-06 | 0.0001007 |
| ROBO1 | 0.816459554 | 4.78521 | 2.89E-06 | 6.25E-05 |
| PBK | 0.81438646 | 3.50071 | 0.0005476 | 0.0039431 |
| CXXC4 | 0.809703569 | 5.16401 | 4.87E-07 | 1.50E-05 |
| AMPH | 0.809438265 | 4.81987 | 2.47E-06 | 5.51E-05 |
| ACYP1 | 0.809196263 | 6.52336 | 3.67E-10 | 4.33E-08 |
| FZD3 | 0.809077867 | 6.00288 | 6.63E-09 | 4.76E-07 |
| TAGLN3 | 0.80805373 | 3.78946 | 0.0001884 | 0.0017104 |
| FAM221A | 0.807917339 | 6.55961 | 2.98E-10 | 3.69E-08 |
| PEG10 | 0.806585746 | 2.46324 | 0.0144297 | 0.0486767 |
| SLAIN1 | 0.80617796 | 4.59205 | 6.90E-06 | 0.0001257 |
| SPC25 | 0.80325962 | 4.1011 | 5.53E-05 | 0.0006589 |
| GPR137C | 0.801884019 | 7.66827 | 3.67E-13 | 1.54E-10 |
| DPY19L2P2 | 0.800600555 | 4.94878 | 1.36E-06 | 3.41E-05 |
| FOXG1 | 0.799217377 | 6.25415 | 1.67E-09 | 1.56E-07 |
| PIMREG | 0.798829722 | 5.51075 | 8.73E-08 | 3.68E-06 |
| DNA2 | 0.798824594 | 6.6668 | 1.61E-10 | 2.21E-08 |
| SCN3A | 0.796288462 | 4.95762 | 1.30E-06 | 3.29E-05 |
| KIAA0895 | 0.791087934 | 6.36236 | 9.15E-10 | 9.13E-08 |
| ZFP69B | 0.790744396 | 6.40088 | 7.36E-10 | 7.61E-08 |
| LOC101928076 | 0.78517329 | 5.19664 | 4.16E-07 | 1.31E-05 |
| CDCA8 | 0.783093906 | 5.73556 | 2.74E-08 | 1.46E-06 |
| PGBD1 | 0.779014122 | 7.12613 | 1.06E-11 | 2.39E-09 |
| UBE2C | 0.778392586 | 3.54199 | 0.000472 | 0.0035386 |
| KCND2 | 0.778004886 | 5.67372 | 3.78E-08 | 1.93E-06 |
| SST | 0.770524288 | 3.18656 | 0.0016192 | 0.0091284 |
| DPP10 | 0.769976089 | 4.14993 | 4.54E-05 | 0.0005634 |
| LRRN1 | 0.769569416 | 3.70656 | 0.0002577 | 0.0021995 |
| FNBP1L | 0.764252683 | 6.43215 | 6.17E-10 | 6.58E-08 |
| ZMAT4 | 0.763465558 | 4.46994 | 1.18E-05 | 0.0001954 |
| RIPPLY3 | 0.762782219 | 5.92476 | 1.01E-08 | 6.54E-07 |
| MYB | 0.761689954 | 5.42303 | 1.36E-07 | 5.38E-06 |
| DSCR8 | 0.761334361 | 4.77804 | 2.99E-06 | 6.41E-05 |
| LHX2 | 0.759461301 | 4.78491 | 2.90E-06 | 6.26E-05 |
| LMNB1 | 0.758667546 | 5.8096 | 1.86E-08 | 1.08E-06 |
| STIL | 0.757281353 | 5.02504 | 9.48E-07 | 2.57E-05 |
| ZNF850 | 0.757113676 | 7.15929 | 8.64E-12 | 2.10E-09 |
| SBK1 | 0.757061276 | 7.38419 | 2.17E-12 | 6.54E-10 |
| CENPV | 0.755148068 | 5.90868 | 1.10E-08 | 6.91E-07 |
| Table S2 continued | | | | |
| Gene | logFC | t | P.Value | adj.P.Val |
| GINS1 | 0.745698131 | 3.68018 | 0.0002844 | 0.0023699 |
| QPCT | 0.742397179 | 3.30607 | 0.0010821 | 0.006729 |
| NUSAP1 | 0.741078234 | 3.94933 | 0.0001015 | 0.0010637 |
| NBEA | 0.741051094 | 3.62822 | 0.0003447 | 0.0027666 |
| YPEL1 | 0.738707303 | 8.20314 | 1.16E-14 | 8.99E-12 |
| UHRF1 | 0.736170578 | 3.45905 | 0.0006352 | 0.0044209 |
| TTK | 0.735048645 | 3.12831 | 0.0019623 | 0.01064 |
| MSH2 | 0.727890539 | 6.75919 | 9.39E-11 | 1.47E-08 |
| ZWINT | 0.727609041 | 4.50575 | 1.01E-05 | 0.0001723 |
| HAUS1 | 0.726221492 | 7.10778 | 1.18E-11 | 2.62E-09 |
| ZNF667-AS1 | 0.723934262 | 3.18706 | 0.0016165 | 0.0091183 |
| ESPL1 | 0.722656862 | 5.26385 | 3.00E-07 | 1.02E-05 |
| C4orf46 | 0.722284656 | 7.42223 | 1.72E-12 | 5.41E-10 |
| TRIT1 | 0.721568207 | 6.36193 | 9.17E-10 | 9.13E-08 |
| SKA1 | 0.720980896 | 4.42949 | 1.40E-05 | 0.0002232 |
| MEX3B | 0.720085223 | 7.61944 | 5.00E-13 | 1.97E-10 |
| CTAG2 | 0.719985033 | 3.83967 | 0.0001555 | 0.0014805 |
| SVBP | 0.719604896 | 8.97713 | 6.25E-17 | 9.69E-14 |
| CHRNA9 | 0.71916785 | 3.71409 | 0.0002505 | 0.0021511 |
| HJURP | 0.717195 | 4.14298 | 4.67E-05 | 0.0005748 |
| MEST | 0.715909707 | 4.64367 | 5.48E-06 | 0.0001038 |
| ERO1B | 0.711473847 | 4.46354 | 1.21E-05 | 0.0001994 |
| GAS2 | 0.711397004 | 2.75848 | 0.0062275 | 0.0258859 |
| C5orf34 | 0.711151932 | 5.46497 | 1.10E-07 | 4.45E-06 |
| SYT13 | 0.71059374 | 2.54537 | 0.0115058 | 0.0410895 |
| ZNF793-AS1 | 0.710363493 | 3.96387 | 9.58E-05 | 0.0010182 |
| RAD51AP1 | 0.710167832 | 3.83176 | 0.0001603 | 0.001517 |
| KIF20A | 0.709485995 | 3.69219 | 0.0002719 | 0.0022907 |
| KIF15 | 0.709439522 | 4.27014 | 2.76E-05 | 0.0003806 |
| POU3F2 | 0.709205182 | 7.0239 | 1.96E-11 | 3.98E-09 |
| AGT | 0.707865964 | 3.01696 | 0.0028115 | 0.0140436 |
| FOXP4-AS1 | 0.703853222 | 5.26936 | 2.92E-07 | 9.93E-06 |
| BTBD3 | 0.70326699 | 5.38766 | 1.62E-07 | 6.14E-06 |
| TYMS | 0.70207109 | 5.66919 | 3.87E-08 | 1.96E-06 |
| FBXO5 | 0.69956876 | 5.21425 | 3.82E-07 | 1.24E-05 |
| MCM2 | 0.698255198 | 3.8093 | 0.0001747 | 0.0016206 |
| DONSON | 0.696163845 | 5.92365 | 1.01E-08 | 6.56E-07 |
| OTULINL | 0.69547503 | 5.50182 | 9.14E-08 | 3.82E-06 |
| MAD2L2 | 0.690963289 | 7.06422 | 1.53E-11 | 3.26E-09 |
| ADAM1A | 0.690909075 | 7.10715 | 1.18E-11 | 2.62E-09 |
| SSX2IP | 0.690662667 | 7.20059 | 6.72E-12 | 1.65E-09 |
| DNALI1 | 0.690513618 | 3.76648 | 0.0002056 | 0.0018351 |
| Table S2 continued | | | | |
| Gene | logFC | t | P.Value | adj.P.Val |
| RAD54L | 0.688849387 | 5.83484 | 1.63E-08 | 9.56E-07 |
| NASP | 0.688781346 | 9.5878 | 8.62E-19 | 4.34E-15 |
| SS18L1 | 0.688199525 | 8.64162 | 6.20E-16 | 8.16E-13 |
| ZSCAN16 | 0.687394072 | 6.17195 | 2.64E-09 | 2.22E-07 |
| BRI3BP | 0.686739729 | 6.51627 | 3.82E-10 | 4.44E-08 |
| MDK | 0.683872099 | 4.30332 | 2.40E-05 | 0.0003407 |
| KDM1A | 0.682852158 | 9.24355 | 9.80E-18 | 2.59E-14 |
| GMNN | 0.682190686 | 5.52814 | 7.99E-08 | 3.41E-06 |
| DLGAP5 | 0.681492453 | 2.79649 | 0.0055596 | 0.023751 |
| TXNDC16 | 0.680803904 | 6.34294 | 1.02E-09 | 9.94E-08 |
| SVIP | 0.678116714 | 4.59006 | 6.96E-06 | 0.0001264 |
| POU4F1 | 0.676039465 | 5.4196 | 1.38E-07 | 5.43E-06 |
| TMPO | 0.675559206 | 7.43354 | 1.60E-12 | 5.12E-10 |
| E2F8 | 0.675515096 | 3.94806 | 0.000102 | 0.0010673 |
| TOP2A | 0.675369607 | 4.1469 | 4.59E-05 | 0.0005694 |
| SMAD9 | 0.674804974 | 4.72922 | 3.73E-06 | 7.72E-05 |
| DEPDC1B | 0.674558632 | 3.64042 | 0.0003295 | 0.0026734 |
| GAS2L3 | 0.673224784 | 5.36724 | 1.80E-07 | 6.71E-06 |
| TPX2 | 0.672578989 | 3.04495 | 0.002571 | 0.0130898 |
| BUB1B | 0.67183325 | 3.52137 | 0.0005085 | 0.003741 |
| PRTFDC1 | 0.671342558 | 4.7494 | 3.41E-06 | 7.15E-05 |
| HENMT1 | 0.670032611 | 4.80176 | 2.68E-06 | 5.88E-05 |
| KCNH2 | 0.669638026 | 3.74678 | 0.0002215 | 0.0019393 |
| NSG2 | 0.669461704 | 4.35365 | 1.94E-05 | 0.0002867 |
| LINC00648 | 0.669009857 | 4.65112 | 5.31E-06 | 0.0001014 |
| UBE2T | 0.668750572 | 4.08166 | 5.99E-05 | 0.0007016 |
| CDCA7L | 0.666409575 | 3.28134 | 0.0011774 | 0.0071688 |
| GPR19 | 0.663970126 | 6.13063 | 3.31E-09 | 2.72E-07 |
| IMMP1L | 0.662835403 | 6.71313 | 1.23E-10 | 1.78E-08 |
| ANXA13 | 0.662467072 | 3.64146 | 0.0003283 | 0.0026663 |
| CRYBA2 | 0.661233536 | 2.98137 | 0.0031471 | 0.0153215 |
| FGF9 | 0.660154953 | 4.31589 | 2.28E-05 | 0.0003275 |
| CCNB1 | 0.659514739 | 3.84249 | 0.0001538 | 0.0014687 |
| SPAG5 | 0.658907756 | 4.07852 | 6.06E-05 | 0.0007085 |
| TEX30 | 0.658123172 | 5.84748 | 1.52E-08 | 9.00E-07 |
| VASH2 | 0.656669438 | 7.54347 | 8.06E-13 | 2.80E-10 |
| WDR76 | 0.656389223 | 6.4775 | 4.77E-10 | 5.23E-08 |
| CENPF | 0.654875475 | 4.98761 | 1.13E-06 | 2.96E-05 |
| TSPAN12 | 0.654550806 | 3.51603 | 0.0005183 | 0.0037996 |
| ZNF670 | 0.654481125 | 7.08999 | 1.31E-11 | 2.88E-09 |
| CLDN11 | 0.65368047 | 5.10385 | 6.51E-07 | 1.91E-05 |
| ITGB3BP | 0.653008521 | 7.46459 | 1.32E-12 | 4.36E-10 |
| Table S2 continued | | | | |
| Gene | logFC | t | P.Value | adj.P.Val |
| ZIK1 | 0.651040828 | 6.82427 | 6.41E-11 | 1.08E-08 |
| LRRC10B | 0.65068364 | 5.33922 | 2.07E-07 | 7.51E-06 |
| RNASEH2A | 0.650574539 | 4.75048 | 3.39E-06 | 7.13E-05 |
| CBLN2 | 0.649069138 | 3.87874 | 0.0001337 | 0.0013198 |
| TRIM9 | 0.649012597 | 5.08776 | 7.03E-07 | 2.03E-05 |
| LOC102724851 | 0.647953742 | 6.70037 | 1.32E-10 | 1.86E-08 |
| EXO1 | 0.646040297 | 3.85357 | 0.0001474 | 0.0014187 |
| DTL | 0.645209537 | 3.40072 | 0.00078 | 0.0051963 |
| PRIM1 | 0.643400545 | 5.20711 | 3.95E-07 | 1.27E-05 |
| PNMA8A | 0.640636242 | 2.46501 | 0.0143603 | 0.0485417 |
| OTUD3 | 0.638179229 | 8.09233 | 2.40E-14 | 1.73E-11 |
| CBFA2T2 | 0.636296821 | 8.42822 | 2.60E-15 | 2.42E-12 |
| RASSF6 | 0.635869814 | 3.85649 | 0.0001457 | 0.0014088 |
| TET1 | 0.634059507 | 5.4095 | 1.45E-07 | 5.67E-06 |
| MYL6B | 0.633500461 | 6.58853 | 2.53E-10 | 3.16E-08 |
| KLK12 | 0.632923117 | 2.64614 | 0.0086478 | 0.0332411 |
| BAALC | 0.632105731 | 3.2039 | 0.0015283 | 0.0087282 |
| RHPN2 | 0.631705452 | 4.0236 | 7.56E-05 | 0.000846 |
| MAD2L1 | 0.62963292 | 3.86521 | 0.0001409 | 0.0013767 |
| TMPO-AS1 | 0.62863541 | 6.09905 | 3.93E-09 | 3.15E-07 |
| INHBE | 0.628492863 | 3.86168 | 0.0001428 | 0.0013882 |
| USP1 | 0.628148005 | 8.18581 | 1.30E-14 | 9.70E-12 |
| KCNMB2 | 0.627880442 | 3.93104 | 0.000109 | 0.0011212 |
| ATAD2 | 0.627761656 | 4.98464 | 1.15E-06 | 2.98E-05 |
| PRKAR2B | 0.6273369 | 3.76409 | 0.0002075 | 0.0018496 |
| ZNF804A | 0.626685235 | 5.76163 | 2.39E-08 | 1.31E-06 |
| EFCAB7 | 0.626318959 | 6.64199 | 1.86E-10 | 2.46E-08 |
| MEX3A | 0.625693399 | 7.98509 | 4.82E-14 | 3.03E-11 |
| RAB39B | 0.62543392 | 3.38742 | 0.000817 | 0.0054005 |
| TMEM150C | 0.625387756 | 4.12115 | 5.10E-05 | 0.0006176 |
| MAP2 | 0.623702107 | 3.23518 | 0.0013763 | 0.0080306 |
| OR7E14P | 0.622095495 | 4.33817 | 2.07E-05 | 0.0003031 |
| EFHD1 | 0.620637552 | 3.47987 | 0.0005899 | 0.0041827 |
| MELK | 0.620231734 | 2.92553 | 0.0037485 | 0.0176136 |
| ELAVL2 | 0.619617271 | 4.89019 | 1.78E-06 | 4.23E-05 |
| PODXL2 | 0.618416434 | 4.9972 | 1.08E-06 | 2.85E-05 |
| RAD54B | 0.617167325 | 4.62818 | 5.88E-06 | 0.0001091 |
| SPATA17 | 0.61693098 | 6.1158 | 3.59E-09 | 2.91E-07 |
| UBE2S | 0.615527983 | 3.60743 | 0.000372 | 0.0029424 |
| CRACD | 0.614938493 | 6.65681 | 1.70E-10 | 2.31E-08 |
| LINC02036 | 0.613344654 | 4.99818 | 1.08E-06 | 2.85E-05 |
| SEPTIN7P13 | 0.613296106 | 5.57537 | 6.28E-08 | 2.86E-06 |
| Table S2 continued | | | | |
| Gene | logFC | t | P.Value | adj.P.Val |
| GDAP1 | 0.612362543 | 4.24591 | 3.05E-05 | 0.0004115 |
| C1orf112 | 0.612219665 | 4.44002 | 1.34E-05 | 0.0002152 |
| ERI3 | 0.610661258 | 7.78535 | 1.74E-13 | 8.78E-11 |
| HMX2 | 0.61032482 | 5.15148 | 5.18E-07 | 1.58E-05 |
| HOOK1 | 0.609169749 | 5.14595 | 5.32E-07 | 1.61E-05 |
| RACGAP1 | 0.608911274 | 3.76405 | 0.0002075 | 0.0018496 |
| KIF18B | 0.603829912 | 3.64684 | 0.0003218 | 0.0026255 |
| CD24 | 0.603302103 | 3.14719 | 0.0018444 | 0.0101039 |
| SOX4 | 0.601952085 | 5.227 | 3.59E-07 | 1.19E-05 |
| ZNF239 | 0.600578152 | 5.19665 | 4.16E-07 | 1.31E-05 |
| ZNF232 | 0.599452087 | 6.95501 | 2.95E-11 | 5.46E-09 |
| CDKN2C | 0.598723989 | 5.29691 | 2.55E-07 | 8.89E-06 |
| PCDH8 | 0.598557778 | 3.50106 | 0.0005469 | 0.0039403 |
| SPAG6 | 0.597168308 | 4.93957 | 1.42E-06 | 3.54E-05 |
| NEFH | 0.596054005 | 3.29209 | 0.001135 | 0.0069742 |
| GCH1 | 0.596005192 | 4.33085 | 2.14E-05 | 0.0003103 |
| FEN1 | 0.595231346 | 4.41508 | 1.49E-05 | 0.0002345 |
| SLC25A21 | 0.594076783 | 3.80245 | 0.0001793 | 0.0016507 |
| UCHL1 | 0.593755489 | 3.18916 | 0.0016053 | 0.0090765 |
| RRM2 | 0.593205333 | 2.78032 | 0.0058352 | 0.0246673 |
| C1orf109 | 0.593185317 | 7.61663 | 5.09E-13 | 1.97E-10 |
| KIF14 | 0.592910793 | 3.18668 | 0.0016186 | 0.0091273 |
| GUSBP14 | 0.591911671 | 5.6001 | 5.53E-08 | 2.56E-06 |
| NARS2 | 0.591578244 | 5.74603 | 2.60E-08 | 1.41E-06 |
| RPRM | 0.591396183 | 3.06744 | 0.0023916 | 0.0124 |
| SETBP1 | 0.591266785 | 4.40901 | 1.53E-05 | 0.0002379 |
| CDH12 | 0.590138248 | 4.43346 | 1.38E-05 | 0.0002197 |
| RCC2 | 0.589527338 | 5.06618 | 7.79E-07 | 2.19E-05 |
| DYNC2I2 | 0.589157963 | 6.59385 | 2.45E-10 | 3.09E-08 |
| ZNF439 | 0.588791881 | 4.99399 | 1.10E-06 | 2.89E-05 |
| AGO3 | 0.588786306 | 8.99483 | 5.53E-17 | 9.29E-14 |
| TRIP13 | 0.587920088 | 3.00248 | 0.0029439 | 0.0145642 |
| FOXA1 | 0.586804933 | 3.3861 | 0.0008208 | 0.0054132 |
| ZNF738 | 0.586032274 | 5.67136 | 3.83E-08 | 1.94E-06 |
| OIP5 | 0.585505635 | 2.94321 | 0.0035476 | 0.0168401 |
| TMEM117 | -0.586410331 | -3.8515 | 0.0001485 | 0.0014273 |
| SLC12A8 | -0.588860551 | -4.2056 | 3.61E-05 | 0.000468 |
| CCL11 | -0.588926341 | -3.3719 | 0.0008623 | 0.0056239 |
| RHOV | -0.589956177 | -3.4106 | 0.0007535 | 0.0050618 |
| RNF135 | -0.590784921 | -4.6799 | 4.66E-06 | 9.18E-05 |
| KCNJ15 | -0.590902536 | -3.8207 | 0.0001672 | 0.0015656 |
| LCE3D | -0.591101042 | -3.39 | 0.0008099 | 0.0053653 |
| Table S2 continued | | | | |
| Gene | logFC | t | P.Value | adj.P.Val |
| EPDR1 | -0.591566569 | -2.9864 | 0.0030972 | 0.0151369 |
| TNFRSF12A | -0.592185542 | -4.8314 | 2.34E-06 | 5.28E-05 |
| ADRB2 | -0.593014067 | -3.8583 | 0.0001447 | 0.0014017 |
| EVA1C | -0.593486174 | -4.4876 | 1.09E-05 | 0.0001839 |
| SESN3 | -0.595203883 | -4.1976 | 3.73E-05 | 0.00048 |
| STEAP2 | -0.595863313 | -4.0318 | 7.32E-05 | 0.0008244 |
| KLK7 | -0.596791522 | -4.8934 | 1.76E-06 | 4.18E-05 |
| ULBP2 | -0.597851015 | -3.0566 | 0.0024766 | 0.0127434 |
| LOC100130691 | -0.59852695 | -4.5398 | 8.69E-06 | 0.0001516 |
| IL36RN | -0.598656187 | -4.3144 | 2.29E-05 | 0.0003287 |
| ABCC3 | -0.600307797 | -5.0766 | 7.42E-07 | 2.12E-05 |
| SLC7A11 | -0.603083021 | -2.4844 | 0.0136187 | 0.0467006 |
| MIR205HG | -0.603757853 | -3.0062 | 0.0029095 | 0.0144328 |
| TM4SF1 | -0.603963432 | -4.097 | 5.63E-05 | 0.0006692 |
| TSPO | -0.605033555 | -5.5719 | 6.39E-08 | 2.89E-06 |
| FAM83A | -0.605928832 | -4.2575 | 2.91E-05 | 0.0003959 |
| RNF39 | -0.606512408 | -6.2459 | 1.75E-09 | 1.61E-07 |
| CAVIN1 | -0.606572312 | -6.5082 | 4.01E-10 | 4.56E-08 |
| ELMO3 | -0.607116462 | -5.5098 | 8.77E-08 | 3.69E-06 |
| LPAR5 | -0.607233362 | -6.0908 | 4.11E-09 | 3.28E-07 |
| REEP6 | -0.607263545 | -5.1437 | 5.37E-07 | 1.63E-05 |
| MVP | -0.608994044 | -5.3979 | 1.54E-07 | 5.93E-06 |
| PRRG4 | -0.610566936 | -4.2449 | 3.07E-05 | 0.0004125 |
| COL10A1 | -0.611551391 | -2.4994 | 0.0130701 | 0.0452271 |
| ORAI3 | -0.613607517 | -7.0479 | 1.69E-11 | 3.56E-09 |
| SEPTIN10 | -0.614502355 | -5.3813 | 1.68E-07 | 6.31E-06 |
| FOS | -0.616267008 | -3.1008 | 0.0021468 | 0.0114113 |
| C16orf54 | -0.616296878 | -3.1109 | 0.0020769 | 0.0111149 |
| ACSL1 | -0.617128403 | -6.0485 | 5.18E-09 | 4.00E-07 |
| RAPGEFL1 | -0.618133142 | -4.2748 | 2.71E-05 | 0.0003748 |
| PLEC | -0.618386447 | -6.2409 | 1.80E-09 | 1.64E-07 |
| TST | -0.618479619 | -4.1073 | 5.40E-05 | 0.0006474 |
| RAB27A | -0.61946414 | -4.4517 | 1.27E-05 | 0.0002065 |
| CA2 | -0.619886997 | -3.124 | 0.0019903 | 0.0107502 |
| MET | -0.620364223 | -4.0325 | 7.30E-05 | 0.0008235 |
| GJB3 | -0.620651776 | -5.2707 | 2.90E-07 | 9.88E-06 |
| ANXA3 | -0.621457582 | -2.5843 | 0.010313 | 0.0378363 |
| CA11 | -0.62203299 | -3.861 | 0.0001432 | 0.001391 |
| TMEM40 | -0.622398773 | -4.363 | 1.86E-05 | 0.0002778 |
| KLK6 | -0.623200726 | -4.3852 | 1.70E-05 | 0.000258 |
| DUOX2 | -0.623625261 | -3.7571 | 0.0002131 | 0.0018849 |
| KLK8 | -0.625965878 | -4.2079 | 3.57E-05 | 0.0004647 |
| Table S2 continued | | | | |
| Gene | logFC | t | P.Value | adj.P.Val |
| LRG1 | -0.626061118 | -3.7167 | 0.0002481 | 0.0021339 |
| PCP4L1 | -0.629465258 | -3.1567 | 0.0017873 | 0.0098475 |
| SGPP2 | -0.629794039 | -3.5659 | 0.000433 | 0.0033025 |
| G6PD | -0.630179863 | -3.7739 | 0.0001999 | 0.001795 |
| SEMA4B | -0.631248684 | -4.8347 | 2.31E-06 | 5.21E-05 |
| SERPINB5 | -0.633083654 | -2.7839 | 0.0057732 | 0.0244305 |
| ITGA3 | -0.639000045 | -4.3147 | 2.29E-05 | 0.0003287 |
| SBSN | -0.64025984 | -3.2302 | 0.0013996 | 0.008141 |
| PYCARD | -0.642934969 | -4.462 | 1.22E-05 | 0.0002005 |
| AIM2 | -0.643514579 | -3.2294 | 0.0014033 | 0.0081541 |
| NECTIN4 | -0.645162122 | -4.8547 | 2.10E-06 | 4.84E-05 |
| CFH | -0.646583078 | -3.6838 | 0.0002805 | 0.0023409 |
| HSPB1 | -0.649834779 | -5.2888 | 2.65E-07 | 9.19E-06 |
| FOXF2 | -0.650568876 | -4.1522 | 4.49E-05 | 0.0005613 |
| CFAP251 | -0.650589924 | -4.439 | 1.35E-05 | 0.0002157 |
| LY6E | -0.651053656 | -3.813 | 0.0001722 | 0.0016013 |
| SLC20A2 | -0.654561125 | -5.791 | 2.05E-08 | 1.16E-06 |
| YAP1 | -0.655265734 | -3.6047 | 0.0003758 | 0.0029653 |
| MMP3 | -0.656871781 | -2.8012 | 0.0054821 | 0.0235244 |
| PPARG | -0.659213779 | -4.7761 | 3.02E-06 | 6.45E-05 |
| SLC7A8 | -0.660695783 | -5.671 | 3.84E-08 | 1.94E-06 |
| MALL | -0.660769228 | -2.6432 | 0.0087206 | 0.0334171 |
| SGK1 | -0.660800075 | -3.5851 | 0.0004036 | 0.0031283 |
| SYTL1 | -0.66265805 | -5.1421 | 5.42E-07 | 1.63E-05 |
| S100A11 | -0.662936704 | -3.6812 | 0.0002833 | 0.0023622 |
| S100A6 | -0.663915499 | -6.9298 | 3.43E-11 | 6.07E-09 |
| EPHA2 | -0.66526865 | -4.176 | 4.08E-05 | 0.0005165 |
| CD44 | -0.666545076 | -6.5234 | 3.67E-10 | 4.33E-08 |
| CDA | -0.667016491 | -3.881 | 0.0001325 | 0.0013102 |
| SLC6A8 | -0.667949008 | -2.9666 | 0.0032973 | 0.0159101 |
| SRPX | -0.672610932 | -4.1343 | 4.83E-05 | 0.0005903 |
| GCNT3 | -0.674006813 | -3.1466 | 0.0018481 | 0.0101188 |
| LRRC8E | -0.676457334 | -5.0205 | 9.68E-07 | 2.62E-05 |
| SOWAHC | -0.676784513 | -4.7888 | 2.85E-06 | 6.17E-05 |
| BLVRB | -0.680411124 | -5.3532 | 1.93E-07 | 7.13E-06 |
| NT5E | -0.683981374 | -4.0804 | 6.02E-05 | 0.0007047 |
| WNT5A | -0.685107227 | -3.1888 | 0.0016074 | 0.0090845 |
| TMEM154 | -0.685989206 | -4.1741 | 4.11E-05 | 0.0005202 |
| TRIM16 | -0.687623942 | -2.9352 | 0.0036375 | 0.0171739 |
| TBC1D2 | -0.689401131 | -5.7247 | 2.90E-08 | 1.54E-06 |
| LINC01133 | -0.691359441 | -4.5364 | 8.82E-06 | 0.0001536 |
| ST6GALNAC1 | -0.691529434 | -2.5455 | 0.0115018 | 0.0410838 |
| Table S2 continued | | | | |
| Gene | logFC | t | P.Value | adj.P.Val |
| RASSF9 | -0.692628646 | -3.4011 | 0.0007788 | 0.0051922 |
| IFI16 | -0.693660148 | -3.9255 | 0.0001114 | 0.00114 |
| HYAL1 | -0.693660404 | -4.2656 | 2.81E-05 | 0.0003868 |
| VSIG10L | -0.693711924 | -4.5163 | 9.63E-06 | 0.0001656 |
| CYP4F2 | -0.695863937 | -3.8164 | 0.00017 | 0.0015835 |
| ETFB | -0.695981162 | -5.449 | 1.19E-07 | 4.78E-06 |
| PTGR1 | -0.698031913 | -4.0117 | 7.93E-05 | 0.0008742 |
| CAV2 | -0.69954925 | -5.9906 | 7.08E-09 | 4.99E-07 |
| DHRS9 | -0.704008173 | -4.3929 | 1.64E-05 | 0.0002507 |
| THBD | -0.705761163 | -4.918 | 1.57E-06 | 3.79E-05 |
| PLS3 | -0.709738468 | -6.9568 | 2.92E-11 | 5.45E-09 |
| MAOA | -0.709809813 | -3.8727 | 0.0001369 | 0.0013441 |
| NPR3 | -0.710603393 | -4.8428 | 2.22E-06 | 5.07E-05 |
| PLAC8 | -0.710722917 | -3.0871 | 0.002244 | 0.0118182 |
| TPSAB1 | -0.711877838 | -4.6169 | 6.18E-06 | 0.0001135 |
| CLDN8 | -0.712097524 | -3.3101 | 0.0010675 | 0.0066621 |
| BTBD11 | -0.71275343 | -3.4167 | 0.0007374 | 0.004982 |
| SQOR | -0.713868667 | -5.3784 | 1.70E-07 | 6.38E-06 |
| S100A10 | -0.716503924 | -4.9704 | 1.23E-06 | 3.13E-05 |
| SLCO1B3 | -0.717403943 | -3.3751 | 0.000853 | 0.0055761 |
| CDH3 | -0.719300042 | -3.0835 | 0.0022704 | 0.0119136 |
| LDAF1 | -0.721159982 | -5.773 | 2.25E-08 | 1.25E-06 |
| SCEL | -0.721293691 | -4.0035 | 8.19E-05 | 0.0008976 |
| CLIP4 | -0.722074909 | -6.7159 | 1.21E-10 | 1.77E-08 |
| EGFL6 | -0.722294786 | -3.9247 | 0.0001118 | 0.0011414 |
| BAG3 | -0.725359463 | -4.7864 | 2.88E-06 | 6.23E-05 |
| CXCL1 | -0.725760689 | -2.8982 | 0.0040796 | 0.0187988 |
| NQO1 | -0.726595036 | -3.0951 | 0.0021869 | 0.0115809 |
| TNFSF10 | -0.728458723 | -3.9103 | 0.0001182 | 0.0011978 |
| AVPI1 | -0.729049297 | -6.4893 | 4.46E-10 | 4.94E-08 |
| SUSD4 | -0.729920377 | -3.1323 | 0.001937 | 0.01054 |
| MYOF | -0.731919218 | -5.633 | 4.67E-08 | 2.25E-06 |
| MMP7 | -0.737631318 | -2.6236 | 0.0092244 | 0.0348246 |
| ZBED2 | -0.739090735 | -3.9194 | 0.0001141 | 0.0011617 |
| RAB7B | -0.741179984 | -5.6807 | 3.65E-08 | 1.87E-06 |
| NAP1L2 | -0.744008472 | -3.4477 | 0.0006611 | 0.0045567 |
| PIGR | -0.74412971 | -2.8581 | 0.0046139 | 0.0206109 |
| AADAC | -0.744651083 | -3.2831 | 0.0011703 | 0.0071409 |
| HCAR3 | -0.74558765 | -2.8861 | 0.0042346 | 0.0193276 |
| GJB5 | -0.746865315 | -3.6877 | 0.0002765 | 0.0023149 |
| CAV1 | -0.747916937 | -5.0528 | 8.31E-07 | 2.32E-05 |
| FRMD6 | -0.749142996 | -3.4841 | 0.000581 | 0.0041403 |
| Table S2 continued | | | | |
| Gene | logFC | t | P.Value | adj.P.Val |
| IFI27 | -0.749388567 | -3.4645 | 0.0006229 | 0.0043575 |
| C3 | -0.750063502 | -2.842 | 0.0048463 | 0.0213836 |
| CX3CL1 | -0.750149767 | -5.5883 | 5.88E-08 | 2.70E-06 |
| TRIP6 | -0.752443683 | -4.6882 | 4.49E-06 | 8.91E-05 |
| PITX1 | -0.752933586 | -3.7806 | 0.0001949 | 0.0017575 |
| GPC1 | -0.754239064 | -4.9738 | 1.21E-06 | 3.10E-05 |
| VTCN1 | -0.757009475 | -3.653 | 0.0003145 | 0.0025832 |
| C1orf116 | -0.758545968 | -3.3661 | 0.0008799 | 0.0057139 |
| CD109 | -0.764532683 | -4.7498 | 3.40E-06 | 7.14E-05 |
| SDC1 | -0.765271953 | -4.1679 | 4.21E-05 | 0.0005315 |
| DPP4 | -0.765358824 | -3.131 | 0.0019449 | 0.0105687 |
| MMP13 | -0.766600569 | -3.3196 | 0.0010329 | 0.0064932 |
| GNA15 | -0.767077029 | -4.7135 | 4.01E-06 | 8.19E-05 |
| FAT2 | -0.77069327 | -3.4623 | 0.0006278 | 0.0043824 |
| CERS3 | -0.773651076 | -4.2729 | 2.73E-05 | 0.0003769 |
| PRNP | -0.776291889 | -6.1651 | 2.74E-09 | 2.27E-07 |
| DKK1 | -0.776335014 | -2.5785 | 0.0104855 | 0.0382776 |
| MFAP5 | -0.776622687 | -3.8982 | 0.0001239 | 0.0012436 |
| CCND1 | -0.77705588 | -4.2755 | 2.70E-05 | 0.0003739 |
| FAM83B | -0.777263511 | -3.3303 | 0.000996 | 0.0063121 |
| SLC9A3R1 | -0.779289619 | -5.3091 | 2.40E-07 | 8.46E-06 |
| SNAI2 | -0.779751146 | -3.6777 | 0.000287 | 0.002389 |
| S100A8 | -0.781281289 | -3.1144 | 0.0020533 | 0.0110269 |
| FOXE1 | -0.781309729 | -4.5843 | 7.14E-06 | 0.0001284 |
| LAD1 | -0.782611417 | -5.1739 | 4.64E-07 | 1.44E-05 |
| PTK6 | -0.78273107 | -6.5557 | 3.05E-10 | 3.73E-08 |
| TSPAN1 | -0.783763243 | -4.4907 | 1.08E-05 | 0.0001822 |
| LYPD3 | -0.783896308 | -3.5297 | 0.0004934 | 0.0036564 |
| UPP1 | -0.784190105 | -5.6668 | 3.92E-08 | 1.97E-06 |
| SRXN1 | -0.785237953 | -3.9601 | 9.73E-05 | 0.0010314 |
| CH25H | -0.789526242 | -3.9743 | 9.19E-05 | 0.000988 |
| MN1 | -0.790823458 | -4.6345 | 5.71E-06 | 0.0001066 |
| ADORA2B | -0.791159697 | -4.258 | 2.90E-05 | 0.0003952 |
| CRYBG1 | -0.794896278 | -4.0791 | 6.05E-05 | 0.0007076 |
| LAMC2 | -0.798877183 | -4.2228 | 3.36E-05 | 0.0004444 |
| LTF | -0.804409711 | -2.6319 | 0.009009 | 0.0342559 |
| TMPRSS11E | -0.814543762 | -3.442 | 0.0006746 | 0.0046419 |
| STEAP1 | -0.815682132 | -3.7946 | 0.0001848 | 0.0016893 |
| AKR1C1 | -0.818224302 | -4.0959 | 5.65E-05 | 0.0006711 |
| HLF | -0.820896957 | -4.4853 | 1.10E-05 | 0.0001856 |
| PLAU | -0.822449245 | -3.692 | 0.0002721 | 0.0022907 |
| VSNL1 | -0.827048129 | -2.8243 | 0.0051122 | 0.0223175 |
| Table S2 continued | | | | |
| Gene | logFC | t | P.Value | adj.P.Val |
| CBR1 | -0.828625617 | -4.7058 | 4.15E-06 | 8.37E-05 |
| CA12 | -0.83452592 | -4.6246 | 5.97E-06 | 0.0001105 |
| OSMR | -0.844704725 | -4.2212 | 3.38E-05 | 0.0004468 |
| SFRP4 | -0.844903537 | -3.6685 | 0.000297 | 0.002461 |
| DMKN | -0.849993012 | -4.063 | 6.45E-05 | 0.0007484 |
| ABCA12 | -0.851538789 | -4.155 | 4.44E-05 | 0.0005556 |
| HAS3 | -0.853697311 | -5.5412 | 7.48E-08 | 3.26E-06 |
| CYP2C18 | -0.855713054 | -4.6415 | 5.54E-06 | 0.0001045 |
| SFRP2 | -0.858001908 | -3.0111 | 0.002864 | 0.0142424 |
| TMPRSS11D | -0.858856435 | -3.8502 | 0.0001493 | 0.0014317 |
| GRHL3 | -0.859519902 | -4.0542 | 6.69E-05 | 0.0007694 |
| ITGA2 | -0.868227738 | -4.9212 | 1.55E-06 | 3.75E-05 |
| SERPINB13 | -0.868630186 | -4.039 | 7.11E-05 | 0.0008067 |
| COL17A1 | -0.869891073 | -4.1429 | 4.67E-05 | 0.0005748 |
| DGKA | -0.872690685 | -6.7433 | 1.03E-10 | 1.59E-08 |
| RYR1 | -0.874361288 | -4.0579 | 6.59E-05 | 0.0007592 |
| FOSL1 | -0.880754122 | -5.7295 | 2.83E-08 | 1.50E-06 |
| RHCG | -0.881084961 | -3.5633 | 0.000437 | 0.0033244 |
| ST6GALNAC2 | -0.881589095 | -4.1349 | 4.82E-05 | 0.0005893 |
| SPINK5 | -0.882597094 | -3.949 | 0.0001016 | 0.0010643 |
| TRIM29 | -0.885041783 | -4.3016 | 2.42E-05 | 0.0003422 |
| MPZL2 | -0.88567045 | -4.7311 | 3.70E-06 | 7.68E-05 |
| CPA3 | -0.889237163 | -3.8087 | 0.0001751 | 0.0016228 |
| C10orf99 | -0.889755258 | -4.0299 | 7.37E-05 | 0.0008291 |
| TCN1 | -0.902495194 | -3.6383 | 0.0003321 | 0.0026891 |
| INAVA | -0.902960561 | -3.8986 | 0.0001237 | 0.0012424 |
| NTRK2 | -0.90544657 | -4.3787 | 1.74E-05 | 0.0002627 |
| APOD | -0.90697227 | -4.0737 | 6.18E-05 | 0.0007208 |
| ENDOD1 | -0.90743753 | -7.9711 | 5.27E-14 | 3.13E-11 |
| BNC1 | -0.912928664 | -4.0766 | 6.11E-05 | 0.0007134 |
| TPSB2 | -0.91777201 | -4.5747 | 7.45E-06 | 0.0001326 |
| TP63 | -0.918140734 | -4.1043 | 5.46E-05 | 0.000654 |
| AHNAK2 | -0.930663144 | -6.9423 | 3.18E-11 | 5.68E-09 |
| ADH1C | -0.934328399 | -3.1754 | 0.0016801 | 0.009398 |
| DAPL1 | -0.935654919 | -2.4992 | 0.0130772 | 0.0452439 |
| SLC25A43 | -0.945589993 | -7.3865 | 2.14E-12 | 6.54E-10 |
| CXCL14 | -0.95152806 | -3.9752 | 9.16E-05 | 0.0009856 |
| IVL | -0.954069054 | -4.3059 | 2.37E-05 | 0.0003378 |
| PDZK1IP1 | -0.957423327 | -3.9302 | 0.0001094 | 0.0011232 |
| TENM2 | -0.964365061 | -3.3079 | 0.0010752 | 0.0066927 |
| SFN | -0.970306321 | -3.9106 | 0.0001181 | 0.001197 |
| KLK10 | -0.984847509 | -5.3426 | 2.03E-07 | 7.43E-06 |
| Table S2 continued | | | | |
| Gene | logFC | t | P.Value | adj.P.Val |
| GPAT3 | -0.985125783 | -5.0097 | 1.02E-06 | 2.73E-05 |
| SOST | -0.993791498 | -4.0034 | 8.19E-05 | 0.0008977 |
| SERPINB2 | -0.996373989 | -3.2579 | 0.001275 | 0.0075844 |
| S100A14 | -1.017169787 | -4.1122 | 5.29E-05 | 0.000637 |
| CBR3 | -1.018124877 | -5.4213 | 1.37E-07 | 5.41E-06 |
| RAB38 | -1.022312766 | -4.8755 | 1.91E-06 | 4.46E-05 |
| KLF5 | -1.023519812 | -5.1986 | 4.12E-07 | 1.31E-05 |
| S100A9 | -1.024490759 | -3.5456 | 0.0004659 | 0.0035023 |
| IL36G | -1.033056711 | -4.106 | 5.42E-05 | 0.0006501 |
| ANXA1 | -1.051041286 | -5.6205 | 4.98E-08 | 2.37E-06 |
| S100A16 | -1.054330205 | -5.9841 | 7.34E-09 | 5.14E-07 |
| CYP4F3 | -1.055655704 | -3.1428 | 0.0018711 | 0.0102283 |
| MMP1 | -1.063578988 | -3.0528 | 0.0025071 | 0.0128486 |
| LAMB3 | -1.065812786 | -4.5957 | 6.79E-06 | 0.0001238 |
| PPL | -1.071175502 | -6.7162 | 1.21E-10 | 1.77E-08 |
| KRTDAP | -1.083379264 | -3.9666 | 9.48E-05 | 0.0010109 |
| SLPI | -1.089057849 | -4.2255 | 3.32E-05 | 0.0004406 |
| AKR1C3 | -1.114851091 | -3.2617 | 0.0012583 | 0.0075174 |
| AKR1C2 | -1.122604342 | -4.1537 | 4.47E-05 | 0.0005583 |
| DSC3 | -1.148285449 | -3.2297 | 0.001402 | 0.0081499 |
| PKP1 | -1.150300795 | -4.0579 | 6.59E-05 | 0.0007592 |
| LY6D | -1.156709887 | -3.8035 | 0.0001786 | 0.0016465 |
| FAM110C | -1.176681765 | -5.2798 | 2.77E-07 | 9.48E-06 |
| FXYD3 | -1.18572144 | -6.1187 | 3.53E-09 | 2.90E-07 |
| PTHLH | -1.187172273 | -3.8179 | 0.000169 | 0.0015793 |
| CAPNS2 | -1.193155316 | -4.7169 | 3.95E-06 | 8.10E-05 |
| ADIRF | -1.221059065 | -6.4725 | 4.91E-10 | 5.35E-08 |
| CLDN1 | -1.22959879 | -4.1384 | 4.75E-05 | 0.0005826 |
| FOXQ1 | -1.242337731 | -4.0387 | 7.11E-05 | 0.0008067 |
| SERPINB3 | -1.245765251 | -3.4809 | 0.0005877 | 0.0041748 |
| GBP6 | -1.258555231 | -5.0754 | 7.46E-07 | 2.12E-05 |
| CES1 | -1.259888665 | -4.2435 | 3.08E-05 | 0.0004141 |
| KRT15 | -1.261438935 | -3.2443 | 0.0013346 | 0.0078449 |
| CYP4F11 | -1.263499829 | -4.7101 | 4.07E-06 | 8.27E-05 |
| NTS | -1.297229775 | -2.7344 | 0.0066871 | 0.027323 |
| UPK1B | -1.309401389 | -3.5237 | 0.0005041 | 0.003716 |
| ZNF750 | -1.320904293 | -4.5225 | 9.37E-06 | 0.0001617 |
| C19orf33 | -1.322723119 | -4.6561 | 5.19E-06 | 9.99E-05 |
| TPRG1 | -1.323576642 | -4.8343 | 2.31E-06 | 5.22E-05 |
| S100A7 | -1.324161705 | -3.7272 | 0.0002385 | 0.0020665 |
| IL20RB | -1.336295102 | -4.9232 | 1.53E-06 | 3.73E-05 |
| LCN2 | -1.348890533 | -4.3836 | 1.71E-05 | 0.0002592 |
| Table S2 continued | | | | |
| Gene | logFC | t | P.Value | adj.P.Val |
| CYP2S1 | -1.351844144 | -6.731 | 1.11E-10 | 1.65E-08 |
| TMPRSS4 | -1.439170129 | -5.8499 | 1.50E-08 | 8.93E-07 |
| KRT13 | -1.441330432 | -3.353 | 0.0009209 | 0.0059179 |
| KRT5 | -1.543367859 | -3.085 | 0.0022595 | 0.011878 |
| CSTA | -1.569286261 | -4.4549 | 1.26E-05 | 0.0002043 |
| PTPRZ1 | -1.580620887 | -4.2074 | 3.58E-05 | 0.000465 |
| KRT16 | -1.600178704 | -4.6728 | 4.81E-06 | 9.41E-05 |
| ADH7 | -1.667438606 | -5.3163 | 2.31E-07 | 8.26E-06 |
| NMRAL2P | -1.667820214 | -4.7559 | 3.31E-06 | 6.98E-05 |
| FGFBP1 | -1.676197582 | -5.8556 | 1.46E-08 | 8.72E-07 |
| KRT14 | -1.678745901 | -3.6891 | 0.0002751 | 0.0023077 |
| PI3 | -1.712033166 | -5.1829 | 4.45E-07 | 1.39E-05 |
| GPR87 | -1.729791559 | -5.1297 | 5.75E-07 | 1.72E-05 |
| ALDH3A1 | -1.73152684 | -5.1275 | 5.81E-07 | 1.74E-05 |
| GJB2 | -1.736517529 | -5.0095 | 1.02E-06 | 2.73E-05 |
| S100A2 | -1.738427058 | -4.4164 | 1.48E-05 | 0.0002341 |
| SPRR1A | -1.740701365 | -4.4359 | 1.36E-05 | 0.0002181 |
| CLCA2 | -1.851407793 | -4.9057 | 1.66E-06 | 3.98E-05 |
| KRT6B | -1.902359231 | -5.005 | 1.04E-06 | 2.78E-05 |
| DSG3 | -1.945096376 | -4.5065 | 1.00E-05 | 0.0001721 |
| AKR1B10 | -2.027114357 | -4.5802 | 7.27E-06 | 0.00013 |
| SPRR3 | -2.062224438 | -4.8795 | 1.88E-06 | 4.42E-05 |
| GJB6 | -2.228242426 | -5.3046 | 2.45E-07 | 8.64E-06 |
| SPRR1B | -2.551103383 | -5.538 | 7.60E-08 | 3.30E-06 |
| KRT6A | -2.964819957 | -5.549 | 7.19E-08 | 3.18E-06 |
| logFC, log-transformed fold change; t, t-test statistic | | | | |
