# Supplementary material for: RBBP4: A novel diagnostic and prognostic biomarker for non‐small‐cell lung cancer correlated with autophagic cell death
Source: Cancer Med. 2024 Aug 7;13(15):e70090. doi: 10.1002/cam4.70090 (PMC11304277; doi:10.1002/cam4.70090)
Supplement: Supplementary file 5 — Table S3. [file CAM4-13-e70090-s002.docx]

| Table S3 325 Key DEGs-Autophagy gene relationship pairs | | | |
| --- | --- | --- | --- |
| key DEGs | autophagy | r | P.Value |
| MYC | MYC | 1 | 0 |
| CSF1R | DYNLL2 | 0.988637348 | 0.000192931 |
| CALM1 | SESN1 | 0.983460728 | 0.000408059 |
| DPP4 | ATG9B | 0.980177163 | 0.000585523 |
| CSF1R | STAT3 | 0.975788677 | 0.000872186 |
| ACTA1 | STX12 | 0.972611735 | 0.001114903 |
| CALM1 | ST13 | 0.966698783 | 0.001644992 |
| NOS3 | BCL2L11 | 0.963528691 | 0.001970978 |
| VIM | PRKAB1 | 0.962983291 | 0.002029994 |
| CSF1R | SOGA3 | 0.961551538 | 0.002189008 |
| AC006064 | WDR6 | 0.959712529 | 0.002401926 |
| RRM2 | MAP3K12 | 0.950326045 | 0.003639967 |
| GSTM2 | ATG9B | 0.948786032 | 0.003867142 |
| NOS3 | EI24 | 0.947041986 | 0.004132565 |
| DPP4 | TP53INP1 | 0.945540337 | 0.004368023 |
| CSF1R | DRAM1 | 0.939643738 | 0.005354382 |
| ACTA1 | ATP6V1G1 | 0.939309585 | 0.005413218 |
| CSF1R | IFT20 | 0.937438511 | 0.005748479 |
| NOS3 | CHMP4B | 0.936614219 | 0.005899302 |
| CALM2 | CASP8 | 0.934525813 | 0.006289964 |
| ACTA1 | RAC1 | 0.932924895 | 0.006597717 |
| MMP9 | GDF5 | 0.931994669 | 0.006779835 |
| CALM1 | CTTN | 0.929314345 | 0.007318104 |
| DPP4 | SESN1 | 0.928854957 | 0.007412371 |
| VIM | PI4K2A | 0.928609041 | 0.007463075 |
| MMP9 | CHMP4B | 0.928037089 | 0.007581655 |
| VIM | EIF4EBP1 | 0.925149323 | 0.008194256 |
| CALM1 | STX12 | 0.924441952 | 0.008347847 |
| RRM2 | HAP1 | 0.923910677 | 0.008464115 |
| VIM | NUP93 | 0.921818326 | 0.008929623 |
| NOS3 | CTSA | 0.921645498 | 0.008968616 |
| DPP4 | SCOC | 0.921219752 | 0.009065023 |
| ACTA1 | ST13 | 0.919265228 | 0.009514036 |
| RRM2 | CAMKK2 | 0.918870176 | 0.009606072 |
| VIM | DAP | 0.918144269 | 0.00977631 |
| RRM2 | ANXA7 | 0.915872667 | 0.010318411 |
| CALM2 | VAMP3 | 0.915863084 | 0.010320727 |
| CALM2 | EPM2A | 0.915325491 | 0.01045111 |
| NOS3 | TP73 | 0.914545132 | 0.010641783 |
| NOS3 | HSF2BP | 0.91443923 | 0.010667788 |
| CSF1R | PHYHIP | 0.914394935 | 0.010678674 |
| MYC | HSF2BP | 0.91436329 | 0.010686454 |
| Table S2 continued | | | |
| key DEGs | autophagy | r | P.Value |
| DPP4 | STX12 | 0.912760251 | 0.01108418 |
| CALM2 | PSEN1 | 0.912283609 | 0.011203796 |
| CSF1R | BECN1 | 0.911491737 | 0.011403895 |
| CALM1 | TMEM59 | 0.910852413 | 0.011566697 |
| DPP4 | KCNQ1 | 0.908752648 | 0.012109253 |
| DPP4 | SLC35B3 | 0.908520142 | 0.012170069 |
| MMP9 | EEF1A2 | 0.907313941 | 0.012487939 |
| CSF1R | DHRSX | 0.907073025 | 0.012551902 |
| CSF1R | MAP1LC3C | 0.906224132 | 0.012778542 |
| DPP4 | TNFSF10 | 0.905438092 | 0.012990148 |
| VIM | ITGA3 | 0.904296939 | 0.013300338 |
| AC006064 | PARK7 | 0.901346078 | 0.014118815 |
| MYC | DHRSX | 0.901135273 | 0.014178188 |
| NOS3 | DHRSX | 0.900211069 | 0.014439906 |
| CSF1R | UCHL1 | 0.899944283 | 0.014515883 |
| AC006064 | PELP1 | 0.899596228 | 0.014615295 |
| DPP4 | RRAGB | 0.899384458 | 0.014675941 |
| DPP4 | ST13 | 0.898178356 | 0.015023645 |
| CALM1 | SLC35B3 | 0.896855993 | 0.015409371 |
| CALM1 | CSNK2A2 | 0.896834576 | 0.015415657 |
| DPP4 | CASP1 | 0.895514687 | 0.015805429 |
| ACTA1 | CTTN | 0.895270889 | 0.015877936 |
| CSF1R | LZTS1 | 0.894858585 | 0.016000921 |
| CSF1R | MT3 | 0.894858585 | 0.016000921 |
| CSF1R | SCN1A | 0.894858585 | 0.016000921 |
| CSF1R | SLC22A3 | 0.894858585 | 0.016000921 |
| NOS3 | GBA | 0.891888586 | 0.01690031 |
| ACTA1 | SESN1 | 0.891438038 | 0.01703881 |
| GSTM2 | CLVS1 | 0.8901031 | 0.017452362 |
| GSTM2 | STX12 | 0.889354214 | 0.017686445 |
| GSTM2 | HTR2B | 0.88847961 | 0.017961718 |
| MYC | DRAM1 | 0.887540852 | 0.018259451 |
| VIM | SLC25A19 | 0.887145738 | 0.018385466 |
| VIM | SNRPF | 0.885176132 | 0.019019832 |
| VIM | TOMM5 | 0.883507104 | 0.019565453 |
| CALM1 | RAB11A | 0.882895666 | 0.019767187 |
| MYC | BECN1 | 0.882538447 | 0.019885503 |
| GSTM2 | GRID1 | 0.881755415 | 0.020146038 |
| GSTM2 | SESN1 | 0.879831843 | 0.020792942 |
| VIM | SNRPB | 0.879056317 | 0.021056518 |
| CALM1 | ATG9B | 0.878486293 | 0.021251261 |
| CALM1 | SCOC | 0.878202165 | 0.021348651 |
| Table S2 continued | | | |
| key DEGs | autophagy | r | P.Value |
| CSF1R | CFLAR | 0.877767734 | 0.021497971 |
| NOS3 | ADAMTS7 | 0.875302712 | 0.022354636 |
| AC138811 | VAMP7 | 0.874794855 | 0.022533114 |
| ACTA1 | MYC | 0.873310601 | 0.02305861 |
| CSF1R | HSF2BP | 0.873002547 | 0.0231684 |
| VIM | CISD2 | 0.872421065 | 0.023376315 |
| CALM1 | NLRC4 | 0.87096979 | 0.023899094 |
| ACTA1 | UBQLN2 | 0.870244489 | 0.024162425 |
| ACTA1 | NLRC4 | 0.869000208 | 0.024617378 |
| DPP4 | TMEM59 | 0.868921528 | 0.024646282 |
| MYC | STAT3 | 0.868601439 | 0.024764036 |
| ACTA1 | BCL2L11 | 0.867978633 | 0.02499392 |
| CALM1 | FKBP1B | 0.867951538 | 0.025003943 |
| VIM | HMOX1 | 0.866346671 | 0.025601079 |
| MYC | GDF5 | 0.865363826 | 0.025970081 |
| RRM2 | ATP6V0E1 | 0.864251641 | 0.026390666 |
| CALM1 | RRAGB | 0.864134746 | 0.026435057 |
| CALM1 | TP53INP1 | 0.86323248 | 0.026778889 |
| CALM2 | RRAGC | 0.863001961 | 0.026867073 |
| CALM1 | PDK4 | 0.862950302 | 0.026886853 |
| ACTA1 | BECN1 | 0.862276772 | 0.027145386 |
| MYC | SOGA3 | 0.859955767 | 0.02804528 |
| MMP9 | HSF2BP | 0.859596369 | 0.028185869 |
| CALM2 | RAB8A | 0.859167808 | 0.028353947 |
| VIM | EIF4G1 | 0.858778293 | 0.028507123 |
| MMP9 | ATP6V0B | 0.858449193 | 0.028636846 |
| VIM | CHST3 | 0.857858311 | 0.028870456 |
| RRM2 | PPY | 0.857376417 | 0.029061642 |
| VIM | PARP1 | 0.856327817 | 0.029479725 |
| MMP9 | DHRSX | 0.856221935 | 0.029522099 |
| NOS3 | NHLRC1 | 0.855940306 | 0.029634943 |
| CALM1 | LAMTOR3 | 0.854787211 | 0.030099098 |
| MMP9 | WDR45 | 0.854277565 | 0.030305332 |
| DPP4 | HTR2B | 0.854079704 | 0.030385578 |
| NOS3 | PSAP | 0.853130938 | 0.030771761 |
| DPP4 | CLVS1 | 0.851824695 | 0.031307219 |
| CALM2 | MYLK | 0.850931375 | 0.031675922 |
| MMP9 | TMEM150A | 0.848233731 | 0.032801685 |
| RRM2 | ITGB4 | 0.847763219 | 0.032999934 |
| ACTA1 | ATG9B | 0.847716441 | 0.033019674 |
| CSF1R | MYC | 0.846392057 | 0.03358088 |
| CSF1R | ATG13 | 0.845332162 | 0.034033217 |
| Table S2 continued | | | |
| key DEGs | autophagy | r | P.Value |
| NOS3 | DEPP1 | 0.844411118 | 0.03442861 |
| VIM | TEAD4 | 0.844319089 | 0.034468235 |
| CALM1 | EXOC4 | 0.84387268 | 0.034660751 |
| ACTA1 | RALB | 0.84321239 | 0.034946428 |
| GSTM2 | BCL2L11 | 0.842917883 | 0.035074203 |
| VIM | ARSB | 0.842779834 | 0.035134173 |
| DPP4 | NLRC4 | 0.84187238 | 0.035529577 |
| CALM2 | DPF3 | 0.841246854 | 0.035803349 |
| MMP9 | SLC35C1 | 0.838788107 | 0.036889022 |
| DPP4 | GABARAPL3 | 0.838780894 | 0.036892229 |
| MMP9 | MAP1LC3C | 0.838553771 | 0.036993289 |
| AC138811 | CAMKK2 | 0.838475538 | 0.037028129 |
| GSTM2 | PDK4 | 0.83812986 | 0.037182257 |
| DPP4 | PDK4 | 0.838091542 | 0.037199361 |
| DPP4 | VPS26A | 0.838039186 | 0.037222736 |
| MMP9 | EI24 | 0.838036236 | 0.037224054 |
| AC138811 | RUBCN | 0.83782711 | 0.037317492 |
| CSF1R | STOM | 0.837795094 | 0.037331807 |
| DPP4 | FBXO7 | 0.837775789 | 0.03734044 |
| MMP9 | CHMP6 | 0.837619962 | 0.037410156 |
| DPP4 | FGF7 | 0.837432114 | 0.037494278 |
| DPP4 | IL10 | 0.837432114 | 0.037494278 |
| DPP4 | TMEM74 | 0.837432114 | 0.037494278 |
| CALM2 | SAR1A | 0.837230082 | 0.037584853 |
| GSTM2 | DEPP1 | 0.836877974 | 0.037742953 |
| ACTA1 | TMEM59 | 0.835216301 | 0.03849326 |
| VIM | MFN2 | 0.83494224 | 0.038617674 |
| DPP4 | FANCL | 0.834867802 | 0.038651499 |
| CALM1 | CASP1 | 0.834790019 | 0.038686858 |
| DPP4 | RAB11A | 0.832848866 | 0.039574193 |
| CALM2 | ARNT | 0.832008999 | 0.039961029 |
| MYC | ATP6V1G1 | 0.830656507 | 0.040587678 |
| CALM2 | CDKN1B | 0.830564458 | 0.040630492 |
| ACTA1 | STAT3 | 0.830502421 | 0.040659359 |
| VIM | SLC1A4 | 0.829743931 | 0.041013076 |
| ACTA1 | SLC35B3 | 0.829241346 | 0.041248242 |
| ACTA1 | HSF2BP | 0.828898523 | 0.041409014 |
| CSF1R | CHMP4B | 0.828229656 | 0.04172353 |
| MMP9 | CTSA | 0.827824981 | 0.041914357 |
| GSTM2 | TNFSF10 | 0.827698868 | 0.041973909 |
| MYC | RAC1 | 0.827216088 | 0.042202251 |
| CALM1 | ATP6V1G1 | 0.826754053 | 0.042421321 |
| Table S2 continued | | | |
| key DEGs | autophagy | r | P.Value |
| DPP4 | FUNDC2 | 0.826222938 | 0.0426738 |
| CALM1 | TNFSF10 | 0.825996291 | 0.042781756 |
| CALM1 | RAC1 | 0.825969745 | 0.042794408 |
| NOS3 | BOC | 0.825149595 | 0.043186175 |
| CALM2 | SH3BP4 | 0.825051066 | 0.043233352 |
| CALM1 | NRBP2 | 0.824874312 | 0.043318044 |
| NOS3 | EEF1A2 | 0.824506252 | 0.04349465 |
| DPP4 | NRBP2 | 0.824273448 | 0.04360653 |
| ACTA1 | GBA | 0.822253527 | 0.044582869 |
| DPP4 | TNIK | 0.822135146 | 0.044640402 |
| CALM1 | LEPR | 0.821983081 | 0.044714355 |
| CALM1 | TRIM13 | 0.821852617 | 0.044777849 |
| DPP4 | LEPR | 0.821388415 | 0.045004105 |
| GSTM2 | TP53INP1 | 0.821068408 | 0.045160389 |
| ACTA1 | LEPR | 0.820335606 | 0.045519222 |
| NOS3 | RGS19 | 0.820095564 | 0.045637051 |
| MMP9 | TUSC1 | 0.819876732 | 0.045744593 |
| GSTM2 | ST13 | 0.819451479 | 0.045953913 |
| ACTA1 | CD46 | 0.819069413 | 0.046142355 |
| DPP4 | ATP6V1G1 | 0.818882066 | 0.046234889 |
| CALM1 | FYCO1 | 0.818357846 | 0.046494269 |
| AC006064 | TM9SF1 | 0.817287143 | 0.04702614 |
| NOS3 | VAMP8 | 0.817160138 | 0.047089417 |
| RRM2 | RELA | 0.817129333 | 0.04710477 |
| ACTA1 | SOGA3 | 0.816589891 | 0.047374011 |
| DPP4 | CTTN | 0.815504726 | 0.047917787 |
| GSTM2 | GBA | 0.814714344 | 0.048315661 |
| GSTM2 | S100A8 | 0.814490494 | 0.048428624 |
| NOS3 | TOMM22 | 0.814103388 | 0.04862426 |
| CALM1 | KRCC1 | 0.813509979 | 0.048924868 |
| NOS3 | GDF5 | 0.813254145 | 0.049054733 |
| CALM2 | IKBKB | 0.813118141 | 0.049123835 |
| GSTM2 | WIPI1 | -0.811743307 | 0.04982491 |
| DPP4 | ATP6V1G2 | -0.813392112 | 0.048984679 |
| NOS3 | ATP6V0E1 | -0.813795325 | 0.04878021 |
| GSTM2 | EIF4EBP1 | -0.813926932 | 0.048713559 |
| CALM2 | VPS18 | -0.81434337 | 0.048502935 |
| MMP9 | VAMP3 | -0.814469431 | 0.048439259 |
| MYC | UBQLN4 | -0.816352702 | 0.04749262 |
| CALM2 | POLDIP2 | -0.817437538 | 0.046951262 |
| MMP9 | MAPT | -0.818301827 | 0.046522027 |
| AC006064 | RASIP1 | -0.81949403 | 0.045932949 |
| Table S2 continued | | | |
| key DEGs | autophagy | r | P.Value |
| CALM2 | TP73 | -0.820292235 | 0.045540501 |
| AC006064 | RUBCN | -0.820309507 | 0.045532026 |
| DPP4 | MAPK8IP1 | -0.820678253 | 0.045351273 |
| AC138811 | BCL2L11 | -0.823324839 | 0.04406379 |
| ACTA1 | PRKAB1 | -0.823750277 | 0.04385844 |
| DPP4 | HAP1 | -0.824543338 | 0.04347684 |
| CALM2 | TUSC1 | -0.825040049 | 0.043238628 |
| GSTM2 | DAP | -0.825459632 | 0.043037882 |
| RRM2 | BCL2L11 | -0.825810072 | 0.04287055 |
| CALM1 | HAX1 | -0.827729388 | 0.041959494 |
| DPP4 | CAMKK2 | -0.827798458 | 0.041926879 |
| MMP9 | EPM2A | -0.828006653 | 0.041828638 |
| NOS3 | ATP6V0C | -0.828303784 | 0.041688619 |
| CALM2 | MAPK3 | -0.829764351 | 0.041003535 |
| AC138811 | SLC35C1 | -0.830600707 | 0.04061363 |
| CALM1 | VPS39 | -0.83119518 | 0.040337549 |
| CALM2 | DAPK3 | -0.832151788 | 0.039895138 |
| MYC | ATF4 | -0.838944809 | 0.036819375 |
| DPP4 | ITGA3 | -0.8393923 | 0.036620825 |
| CSF1R | ITGB4 | -0.840511152 | 0.036126605 |
| NOS3 | RPS27A | -0.841312587 | 0.035774533 |
| DPP4 | MUL1 | -0.841657311 | 0.035623595 |
| CALM1 | EIF4EBP1 | -0.841812596 | 0.0355557 |
| MYC | VPS39 | -0.84210346 | 0.035428691 |
| RRM2 | ATG9B | -0.842269072 | 0.035356471 |
| AC006064 | REP15 | -0.844496662 | 0.034391797 |
| CALM1 | PRKAB1 | -0.845516956 | 0.033954146 |
| CALM2 | CTSB | -0.845600888 | 0.033918262 |
| CALM2 | PINK1 | -0.845692911 | 0.033878939 |
| AC138811 | WDR45 | -0.846289324 | 0.033624599 |
| CALM2 | ATP6V1E2 | -0.849382608 | 0.032319975 |
| CALM2 | CAPN1 | -0.850045207 | 0.032043685 |
| CALM1 | MUL1 | -0.851463763 | 0.031455942 |
| AC138811 | CHMP4B | -0.855825906 | 0.02968084 |
| AC138811 | EI24 | -0.858113121 | 0.028769604 |
| MMP9 | SAR1A | -0.858233642 | 0.028721961 |
| GSTM2 | TMEM39B | -0.858285649 | 0.028701414 |
| AC006064 | EXOC8 | -0.859140368 | 0.028364725 |
| GSTM2 | HMOX1 | -0.859482045 | 0.028230659 |
| CALM2 | HSPB1 | -0.860580601 | 0.027801652 |
| CALM1 | TEAD4 | -0.862680247 | 0.026990372 |
| DPP4 | BNIP3 | -0.862808247 | 0.026941283 |
| Table S2 continued | | | |
| key DEGs | autophagy | r | P.Value |
| CALM2 | GMIP | -0.865089634 | 0.026073471 |
| NOS3 | EPM2A | -0.866122576 | 0.025684994 |
| CALM2 | FABP1 | -0.866201841 | 0.025655297 |
| CALM2 | PPARGC1A | -0.866201841 | 0.025655297 |
| CALM2 | PRKAG3 | -0.866201841 | 0.025655297 |
| RRM2 | TP73 | -0.866317572 | 0.025611968 |
| CALM2 | MAP1S | -0.86666118 | 0.02548353 |
| CSF1R | ANXA5 | -0.867095496 | 0.025321624 |
| DPP4 | HMOX1 | -0.870742141 | 0.023981597 |
| CALM1 | PI4K2A | -0.870841798 | 0.023945463 |
| MYC | ITGB4 | -0.871209365 | 0.023812414 |
| MYC | VDAC1 | -0.874744462 | 0.022550861 |
| DPP4 | QSOX1 | -0.876006849 | 0.022108298 |
| ACTA1 | BNIP3 | -0.878477464 | 0.021254284 |
| CALM2 | TMEM150A | -0.87879317 | 0.021146313 |
| MYC | ATG16L1 | -0.879855123 | 0.020785054 |
| CALM2 | LENG9 | -0.881262152 | 0.020310991 |
| CALM2 | P4HB | -0.882843558 | 0.019784425 |
| ACTA1 | HMOX1 | -0.883468321 | 0.01957822 |
| MYC | ANXA5 | -0.883993687 | 0.019405622 |
| MMP9 | ATP6V0C | -0.884186053 | 0.019342607 |
| CALM2 | FOS | -0.884365083 | 0.019284049 |
| CALM1 | NUP93 | -0.884580644 | 0.019213655 |
| CALM2 | ATG4D | -0.885077031 | 0.019052023 |
| ACTA1 | MUL1 | -0.886936282 | 0.018452437 |
| CALM2 | CTSD | -0.887108528 | 0.018397355 |
| ACTA1 | NUP93 | -0.88759866 | 0.018241049 |
| CALM1 | SNRPB | -0.887608866 | 0.018237801 |
| CALM2 | ATP6V0B | -0.893852458 | 0.016302953 |
| CALM2 | PSAP | -0.894732307 | 0.01603868 |
| GSTM2 | SQSTM1 | -0.894902949 | 0.015987666 |
| MMP9 | RHEB | -0.896014441 | 0.015657297 |
| NOS3 | CAMKK2 | -0.897653884 | 0.015176067 |
| ACTA1 | ATF4 | -0.898254885 | 0.015001467 |
| ACTA1 | ITGB4 | -0.899537326 | 0.014632151 |
| ACTA1 | ATP6V1G2 | -0.900261824 | 0.014425473 |
| VIM | CSNK2A2 | -0.901484721 | 0.014079832 |
| ACTA1 | SQSTM1 | -0.903912201 | 0.013405715 |
| GSTM2 | ITGA3 | -0.904732164 | 0.013181617 |
| CALM2 | RAB1B | -0.904792879 | 0.013165096 |
| CALM2 | VPS37D | -0.905657502 | 0.012930912 |
| CALM2 | VAMP8 | -0.905768668 | 0.01290095 |
| Table S2 continued | | | |
| key DEGs | autophagy | r | P.Value |
| DPP4 | SQSTM1 | -0.907832422 | 0.012350818 |
| CALM2 | WDR45 | -0.909395805 | 0.01194179 |
| VIM | CTTN | -0.911476058 | 0.011407874 |
| CALM2 | CHMP6 | -0.911637379 | 0.011366964 |
| ACTA1 | VPS39 | -0.911853979 | 0.011312147 |
| ACTA1 | SNRPF | -0.913677575 | 0.010855723 |
| CSF1R | SOGA1 | -0.915263117 | 0.010466289 |
| CALM2 | EEF1A2 | -0.916160278 | 0.01024899 |
| GSTM2 | HAX1 | -0.917533498 | 0.00992067 |
| CALM2 | CHMP4B | -0.918547838 | 0.009681487 |
| CALM1 | ITGA3 | -0.922608415 | 0.008752419 |
| VIM | FKBP1B | -0.922896939 | 0.008688139 |
| CALM2 | LAMTOR4 | -0.925695286 | 0.008076661 |
| CALM2 | CTSA | -0.928744007 | 0.007435227 |
| NOS3 | VPS39 | -0.929134235 | 0.007354993 |
| GSTM2 | CAMKK2 | -0.929791492 | 0.007220815 |
| CALM2 | TOMM22 | -0.93069636 | 0.007038059 |
| CALM1 | DAP | -0.932078936 | 0.006763237 |
| CALM2 | EI24 | -0.939432686 | 0.005391507 |
| CSF1R | ATP6V0E1 | -0.94170866 | 0.004997787 |
| CALM1 | QSOX1 | -0.942714823 | 0.004828394 |
| ACTA1 | QSOX1 | -0.947231303 | 0.004103335 |
| MYC | ATP6V0C | -0.949009805 | 0.003833713 |
| CALM1 | SNRPF | -0.951824012 | 0.003425482 |
| CALM2 | SLC35C1 | -0.959258405 | 0.002456003 |
| AC138811 | TP73 | -0.959324582 | 0.002448086 |
| CALM1 | SQSTM1 | -0.966853183 | 0.001629858 |
| DPP4 | HAX1 | -0.982246137 | 0.000470001 |
| CALM1 | HMOX1 | -0.983254485 | 0.000418271 |
| CSF1R | VDAC1 | -0.984870188 | 0.000341635 |
| CSF1R | ATG16L1 | -0.991390008 | 0.000110879 |
